# Antifungal activity of bio-active cell-free culture extracts and volatile organic compounds (VOCs) synthesised by endophytic fungal isolates of Garden Nasturtium

**DOI:** 10.1038/s41598-024-60948-0

**Published:** 2024-05-16

**Authors:** Hiran Kanti Santra, Riya Dutta, Debdulal Banerjee

**Affiliations:** 1 Microbiology and Microbial Biotechnology Laboratory, Department of Botany and Forestry, Vidyasagar University, Midnapore, West Bengal 721102 India; 2 Center for Life Sciences, Vidyasagar University, Midnapore, West Bengal 721102 India

**Keywords:** *T. majus* endophyte, Anti-Candida, Anti-fungal, VOCs, Biochemistry, Biotechnology, Plant sciences

## Abstract

Antimicrobial resistance in fungal pathogens (both human and plant) is increasing alarmingly, leading to massive economic crises. The existing anti-fungal agents are becoming ineffective, and the situation worsens on a logarithmic scale. Novel antifungals from unique natural sources are highly sought to cope sustainably with the situation. Metabolites from endophytic microbes are the best-fitted alternatives in this case. Endophytes are the untapped sources of ‘plants’ internal microbial population’ and are promising sources of effective bio-therapeutic agents. Fungal endophytes were isolated from *Tropaeolum majus* and checked for antifungal activity against selected plant and human pathogens. Bioactive metabolites were identified through chromatographic techniques. The mode of action of those metabolites was evaluated through various spectroscopic techniques. The production of antifungal metabolite was optimized also. In particular VOCs (volatile organic compounds) of TML9 were tested in vitro for their anti-phytopathogenic activity. Ethyl acetate (EA) extract of cell-free culture components of *Colletotrichum aenigma* TML3 exhibited broad-spectrum antifungal activity against four species of Candida and the major constituents reported were 6-pentyl-2H-pyran-2-one, 2-Nonanone, 1 propanol 2-amino. The volatile metabolites, trans-ocimene, geraniol, and 4-terpinyl acetate, produced from *Curvularia lunata* TML9, inhibited the growth of some selected phyto pathogens. EA extract hampered the biofilm formation, minimised the haemolytic effect, and blocked the transformation of *Candida albicans* (MTCC 4748) from yeast to hyphal form with a Minimum Fungicidal Concentration (MFC) of 200–600 µg mL^−1^. Central carbohydrate metabolism, ergosterol synthesis, and membrane permeability were adversely affected and caused the lethal leakage of necessary macromolecules of *C. albicans*. Volatile metabolites inhibited the growth of phytopathogens i.e., *Rhizoctonia solani*, *Alternaria alternata*, *Botrytis cinerea*, *Cercospora beticola*, *Penicillium digitatum*, *Aspergillus fumigatus*, *Ceratocystis ulmi*, *Pythium ultimum* up to 89% with an IC_50_ value of 21.3–69.6 µL 50 mL^−1^ and caused leakage of soluble proteins and other intracellular molecules. Citrusy sweet odor volatiles of TML9 cultured in wheat-husk minimised the infections of *Penicillium digitatum* (green mold), in VOC-exposed sweet oranges (*Citrus sinensis*). Volatile and non-volatile antifungal metabolites of these two *T. majus* endophytes hold agricultural and pharmaceutical interests. Metabolites of TML3 have strong anti-Candida activity and require further assessment for therapeutic applications. Also, volatile metabolites of TML9 can be further studied as a source of antifungals. The present investigational outcomes bio-prospects the efficacy of fungal endophytes of Garden Nasturtium.

## Introduction

The overexploitation of antibiotics has led to the spreading of antimicrobial resistance in deadly pathogens. If left untackled, this situation may cause a toll of 700,000 people of different age groups yearly, and the number is projected to reach 10 million by 2050^1^. AMR incurs substantial economic burdens, alongside the consequences of mortality and disability. According to the World Bank, antimicrobial resistance (AMR) might lead to an increase of US$ 1 trillion in healthcare expenses by 2050 and annual gross domestic product (GDP) losses ranging from US$ 1 trillion to US$ 3.4 trillion by 2030. The global rise in antibiotic resistance poses a significant threat, diminishing the efficacy of common antibiotics against widespread bacterial infections. The 2022 Global Antimicrobial Resistance and Use Surveillance System (GLASS) report emphasises concerning levels of resistance observed among common microbiological infections. The WHO FPPL (Fungal Pathogen Priority List) includes fungal members that cause invasive acute and subacute infections and lead to massive drug resistance. The different species of *Candida*, i.e., *C. albicans*, *C. krusei*, *C. auris*, and *C. tropicalis,* are placed in critical-priority and high-priority groups, respectively. WHO identifies antibiotic-resistant microorganisms (ARM) and antibiotic-resistant genes (ARG) as the prime public health hazard of the twenty-first century. The One Health Initiative targets improving human health and excavating potent ways/medications to tackle AMR tops the list. Multi-drug resistance can be triggered by several variables, including excessive and irrational use of antibiotics, delayed infection diagnosis, unsanitary living situations, and a decrease in hosts’ immunity. Incredibly opportunistic fungal pathogens like *Candida albicans* and other non-albicans species- *C. tropicalis*, *C. glabrata*, and *C. krusei* cause dreadful infections in immune-compromised patients. Persons infected with HIV and undergoing immunosuppressant therapy or chemotherapy for the treatment of cancer are the worst affected groups^2^. Both superficial infections and invasive pathogenesis create pandemonium for medical personnel to recover the patients and the situation demands high alert^3^. *C. albicans* comes to the list of very common microflora inhabiting multiple tissues like skin, urinogenital tract, and oral cavity, but they can be fatal in immunocompromised conditions. In particular, blood-borne candidiasis and mucocutaneous infections have a severe mortality rate of 47%^4,5^. The treatment options are limiting, and the challenge is severe due to the allied eukaryotic genetic structure and biochemical conditions of humans (mammalian host cells) and invading fungal pathogens^6–8^. The ability of candida cells to form biofilm and transform from yeast to hyphal cells are the two critical factors in its treatment with available antibiotics^9^. The resistance develops very soon as our immune system can’t invade the biofilm^10^. This dimorphic fungus *Candida* has emerged as a fatal pathogen in the population and is becoming multidrug resistant daily. The situation is alarming and demands effective solutions. As the existing agents are getting resistant, it is high time to look for new and novel agents with long-term efficacy. Natural products from novel sources are the most trusted alternatives here. The search focuses mainly on the less explored biological organisms. Nowadays, endosymbiotic microflora of green plants, i.e., endophytic fungi or actinobacteria, contribute to antimicrobial drug discovery^11,12^. Endophytes are ubiquitous in nature and live asymptomatically within the internal tissues of plants, also protecting the plant from various biotic or abiotic stresses. They synthesise diverse bioactive compounds with agricultural and pharmaceutical utility. Alkaloids, peptides, phenolic derivatives, polyketones, steroids, and terpenoids are a few examples; more than 50% of endophyte-synthesized compounds are novel^13^. Endophytes are symbiotic partners of plants and have co-evolved together. As a result, they share valuable plant genes and thus can produce metabolites similar to or superior to plants’ metabolome. These might result from horizontal or vertical gene transfer across the two different but closely associated species^14,15^. Endophytic fungal metabolites are preferred over others due to their easy operational procedures, and diverse utility in agricultural and pharmaceutical sectors for developing antimicrobial, anti-oxidative, anti-proliferative, and plant growth-promoting compounds^16,17^. Endophytes hold multidisciplinary utility but reports of antifungal (particularly against human pathogenic fungus) or anticandidal substances from them are scanty.

Other than human fungal pathogens, phytopathogenic fungi are estimated to cause seventy to eighty percent of total agricultural loss for both standing and post-harvest crops worldwide with wastage of hundreds of billions of dollars yearly^21–23^. Contemporary solutions like chemical pesticides and fungicides are short-term remedies and are developing quick resistance. Other than chemical formulations, with major long-term side effects for ecosystem and human health, novel sustainable solutions are to be sought. Here also, volatile metabolites from endophytes with broad-spectrum anti-phyto-pathogenic activity represent a new domain of bio-based options^24,25^.

Here fungal endophytes of Garden Nasturtium- *Tropaeolum majus* (Family- Tropeolaceae) have been studied to produce effective anti-fungal metabolites. This medicinal herb is traditionally used to treat sepsis, urinary tract infections (UTIs), anemia, and chest colds. It also has expectorant, antibacterial, antifungal, and antioxidant properties^18^. It harbors a rich source of anthocyanins, polyphenols, vitamin C, and necessary minerals- zinc, iron, and copper, especially glucotropeolin, which has increased its pharmaceutical relevance. The endophytic community of this beautiful herb has not been explored except for a recent report about the biofertilizer potentials of root endophytes^20^. Here, we have tried to identify the potent fungal endophytes of Garden Nasturtium for the production of anti-fungal metabolites, along with their characterisation and mode of action to bio-prospect their potent pharmaceutical and agricultural applications.

## Results

### Isolation and identification of fungal endophytes

In the present study, a hundred-three endophytic fungal isolates were obtained from 40 plant segments (i.e., leaf and stem fragments of *Tropaeolum majus*) with a colonization frequency of 73.57%. The fungal endophytes were non-epiphytic, which was confirmed by the tissue fingerprinting method. Briefly, the aliquots with explant wash liquid were plated on PDA and no fungi appeared on the plates. The endophytes were isolated from disease-free plant parts and considered non-pathogenic. The final confirmation was obtained from the results of Koch’s Postulate where the plants infected with endophyte did not show any disease symptoms.

**Figure 1:**
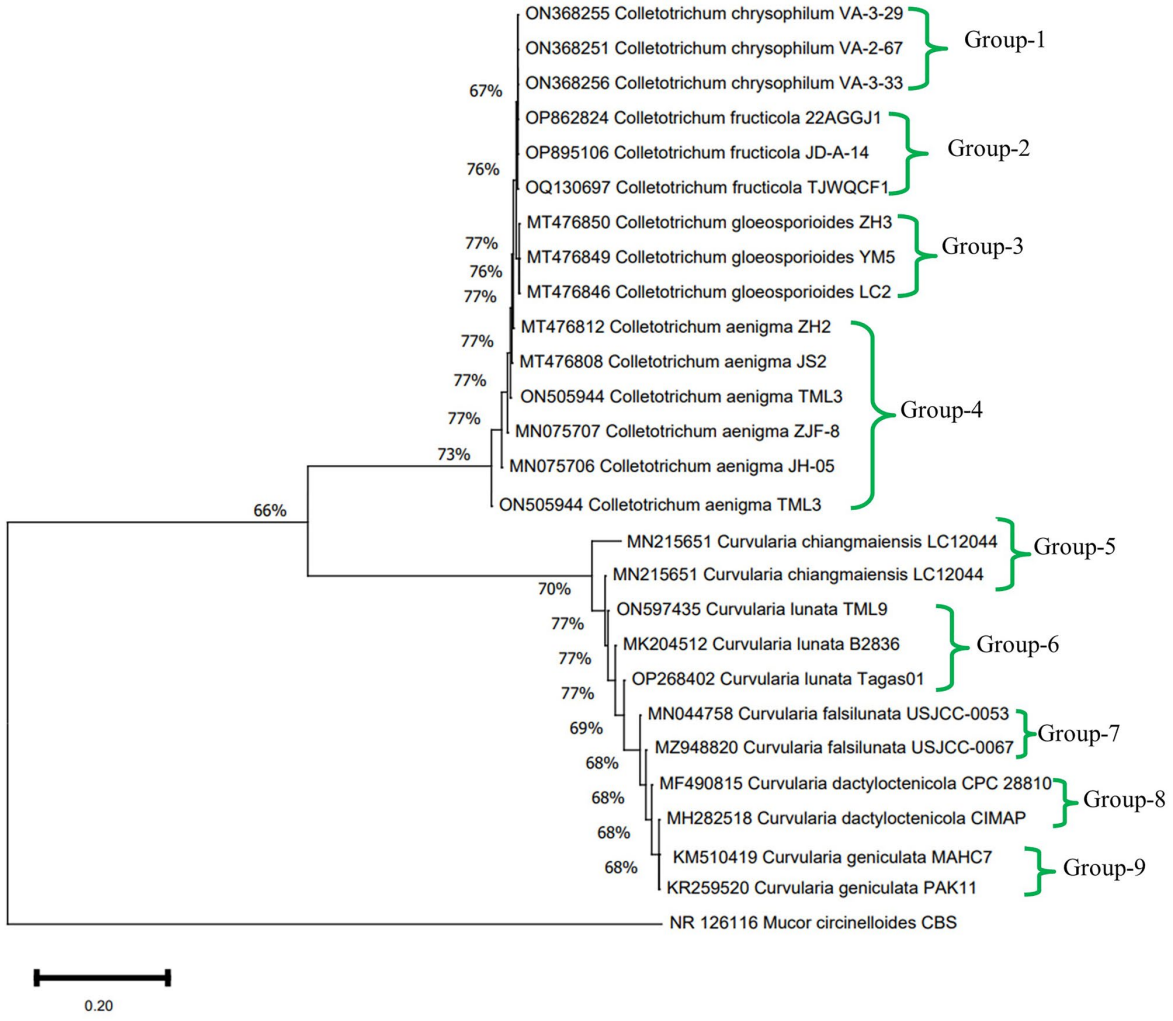
Phylogenetic tree of the endophytic *Colletotrichum aenigma* TML3 and *Curvularia lunata* TML9, isolates of *Tropaeolum majus*.

Seven endophytic fungal isolates were identified (*Penicillium* sp., *Phoma* sp., *Phomopsis* sp., *Nigropora* sp., *Aspergillus* sp., *Pestalotiopsis* sp., *Curvularia* sp.) based on their plate (pigment production, spore formation, growth pattern, hyphal aggregations) and microscopic morphology. Two sterile isolates with the most efficient antifungal activity were identified at the molecular level by amplifying conserved ITS regions (Supplementary Fig. 1 a–d). TML3 and TML9 were identified as *Colletotrichum aenigma* and *Curvularia lunata* with a GenBank Accession number of ON505944 and ON597435, respectively. Five isolates remained unidentified and did not form any spore or other reproductive structure even after inoculation in CLA (carnation leaf pieces agar). Universal primers ITS1 and ITS4 were used to amplify the conserved regions of 28S rDNA and 18S rDNA, ITS1-5.8S-ITS2, respectively. The amplification products were sequenced, and results were confirmed by comparing the sequences against the GenBank database of NCBI using the BLAST tool. Gaps and missing data were removed from the dataset. There were 583 and 540 nucleotides in the final dataset of TML3 and TML9, respectively. A phylogenetic tree illustrated the sequence of TML3 and TML9 with the existing fungal nucleotide database in GenBank (Fig. 1). The neighbor-joining method was adopted to analyze evolutionary distances, and the phylogenetic tree was constructed by bootstrap analysis. The tree depicts that the endophytic fungal isolate TML3 and TML9 is phylogenetically related to the *Colletotrichum aenigma* and *Curvularia lunata*, and it is supported by high bootstrap values (77%) and matching of query coverage (100%). Cladograms were constructed by performing five hundred bootstrapping repetitions. The phylogram was divided into nine groups. This grouping was based on different species of *Curvularia* and *Colletotrichum* involved in the tree preparation with a high to low similarity index. The total branch length of the phylogenetic tree was calculated as 0.5182275222.

### Anti-fungal activity of the endophytic fungal isolates

#### Anti-candida activity of fungal metabolites

The cell-free culture extract of endophytic isolate- *C. aenigma* TML3 exhibited maximum anti-candida (10.66 mm clear zone of inhibition) activity against *Candida albicans*. In-vivo anti-Candida activity was also assessed, and the metabolites effectively controlled infection in the kidney tissues of the male Wistar rats (Supplementary Fig. 2). In vitro, gene expression analysis of the treated *C. albicans* cells confirms that the virulence-related genes- *Sap1*, *Erg3*, and *Erg11* are downregulated upon exposure to TML3 metabolites (Supplementary Fig. 3).

#### Volatile antifungal activity of endophytic isolate

The antifungal activity of the endophytic isolate was checked against nine fungal pathogens mentioned in the materials and method section. The gas test was done by split-plate method to evaluate the volatile antifungal production ability of the isolates, and only one isolate synthesize volatile antifungals with a characteristic odor. VOCs of the isolate *C. lunata* TML9 inhibited the growth of fungal pathogens drastically up to 89% (Table 1). A six-day exposure causes the complete cessation of pathogens’ growth, but an exposure of four days can restrict the pathogen’s growth up to 89%.

**Table 1:** Anti-fungal activity of a six-day-old endophytic fungus *Curvularia lunata* TML9 against selected fungal pathogens.

Fungal pathogens	Inhibition (%) after 96 h of treatment^a^	IC_50_ of artificial atmosphere after 72 h of treatment (µL 50 mL^−1^)	IC_50_ as µL mL^−1^ of air space needed for 50% inhibition^b^
*Rhizoctonia solani* (MTCC-4634)	43.5 ± 0.9	53.7 ± 1.9	1.07
*Alternaria alternata* (MTCC-3793)	57.4 ± 1.3	27.8 ± 0.6	0.56
*Geotrichum candidum* (MTCC-3993)	No inhibition	69.6 ± 2.2	1.39
*Botrytis cinerea* (MTCC-8659)	59.8 ± 0.9	29.5 ± 2	0.59
*Cercospora beticola* (ATCC-12825)	67.5 ± 1.5	32.0 ± 1	0.64
*Penicillium digitatum* (ATCC-34644)	77.8 ± 0.8	24.5 ± 0.5	0.49
*Aspergillus fumigatus* (MTCC-3785)	59.7 ± 1	38.6 ± 0.8	0.77
*Ceratocystis ulmi* (ATCC-32437)	23.6 ± 0.3	47.8 ± 0.9	0.96
*Pythium ultimum* (ATCC-200006)	88.9 ± 1.7	21.3 ± 1.2	0.43

^a^Antifungal action was calculated as a percentage of pathogenic fungal growth inhibition relative to the growth of the same pathogenic fungi under control conditions.

^b^Concentration of VOCs’ artificial mixture (prepared with standards) in microliters per millilitre of air space required to produce a 50% reduction of the pathogenic test fungi.

### Mode of action of the antifungal metabolites

#### The minimum fungicidal concentration of endophytic isolates

EA extract of endophytic isolate (TML3) was lethal against all four candida pathogens- *C. albicans*, *C. tropicalis*, *C. glabrata*, and *C. krusei* at a minimum inhibitory and fungicidal concentration range of 200 µg mL^−1^ to 600 µg mL^−1^ (Table 2).

**Table 2:** Anticandidal activity of EA extract of *C. aenigma* TML3 (µg mL^−1^).

Pathogenic microorganisms	TML3	
*Candida albicans* (MTCC 4748)	MIC	MFC
	200	300
*C. glabrata* (ATCC 2001)	300	400
*C. krusei* (ATCC 6258)	250	350
*C. tropicalis* (MTCC 184)	350	600

MIC-minimum inhibitory concentration. MFC-minimum fungicidal concentration.

#### Fungal metabolites cause leakage of biomacromolecules from candida cells

**Figure 2:**
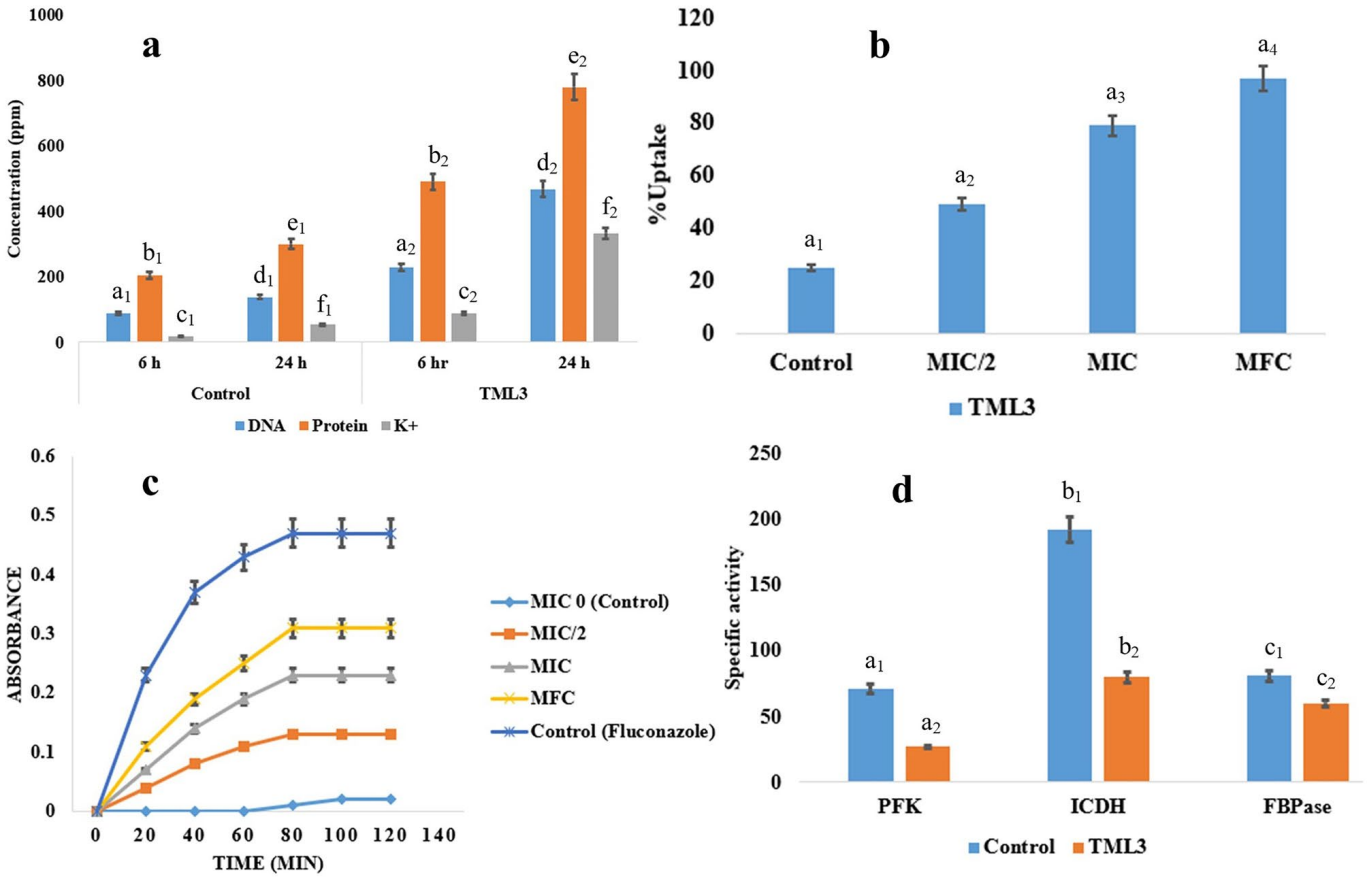
Effect of *C. aenigma* TML3 metabolites on *C. albicans* in terms of (**a**) Leakage of necessary biomacromolecules, (**b**) Alteration in the uptake of crystal violet, (**c**) release of a valuable enzyme- cytoplasmic β- galactosidase and (**d**) decrease in specific activity of enzymes involved in carbohydrate metabolism. Values are the three replicates' means ± standard error (SE). The different letters indicate a valid statistical difference between the sample and the control set at P < 0.05 level (analysed by Tukey’s multiple comparison test).

Necessary intracellular biomacromolecules like DNA, protein, and K^+^ ions were found to be leaked in the extracellular environment after continuous treatment for 24 h with TML3 metabolites. The leakage indicates the cidal action of the fungal metabolites against *Candida* pathogens. There was a two-to-four-fold increase (P < 0.001) of DNA, protein, and K^+^ ion contents after 24 h of treatment in comparison to the 6 h treatment (Fig. 2a). This leakage causes the loss of necessary elements of candida pathogens and facilitates the penetration of other endophytic fungal metabolites.

#### Fungal metabolites cause membrane damage to *C. albicans*

Crystal violet uptake is a significant marker for detecting the condition of the candida cell membrane. Damage caused by fungal metabolites on the candida cell membrane allows the inflow of crystal violet. The penetrance of the stain indicates membrane damage. TML3 metabolites at MFC doses exhibited maximum membrane damage i.e., higher uptake of crystal violet stain compared to fluconazole. Crystal violet uptake increased from 24.68% (control-untreated one) to 96.72% (Fig. 2b) after treatment with MFC doses of TML3 metabolites.

#### Endophytic metabolites block the central carbohydrate metabolism of *C. albicans*

Further confirmation of candida cell membrane damage by endophytic fungal metabolites was evaluated by checking the levels of released β-galactosidase in the surrounding liquid extract leaked due to membrane disruption and hampering the energy utilization. A higher amount of leakage of the necessary enzyme β-galactosidase is reported when the TML3 extract was applied at MFC dose (0.02–0.31 unit of absorbance), and also a mild increase in leakage of the enzyme at MIC doses (0.02–0.23 unit of absorbance) is noted (Fig. 2c).

Fungal metabolites directly hamper the central carbohydrate metabolism of pathogenic cells. The three most physiologically active enzymes (PFK, ICDH, and FBPase) involved in the energy generation procedure interfered drastically. There was a dose-dependent response, and at MFC doses, the candida cells were drastically affected (Fig. 2d).

#### Inhibition of hyphal development and arrest of *C. albicans* in its yeast form

**Figure 3:**
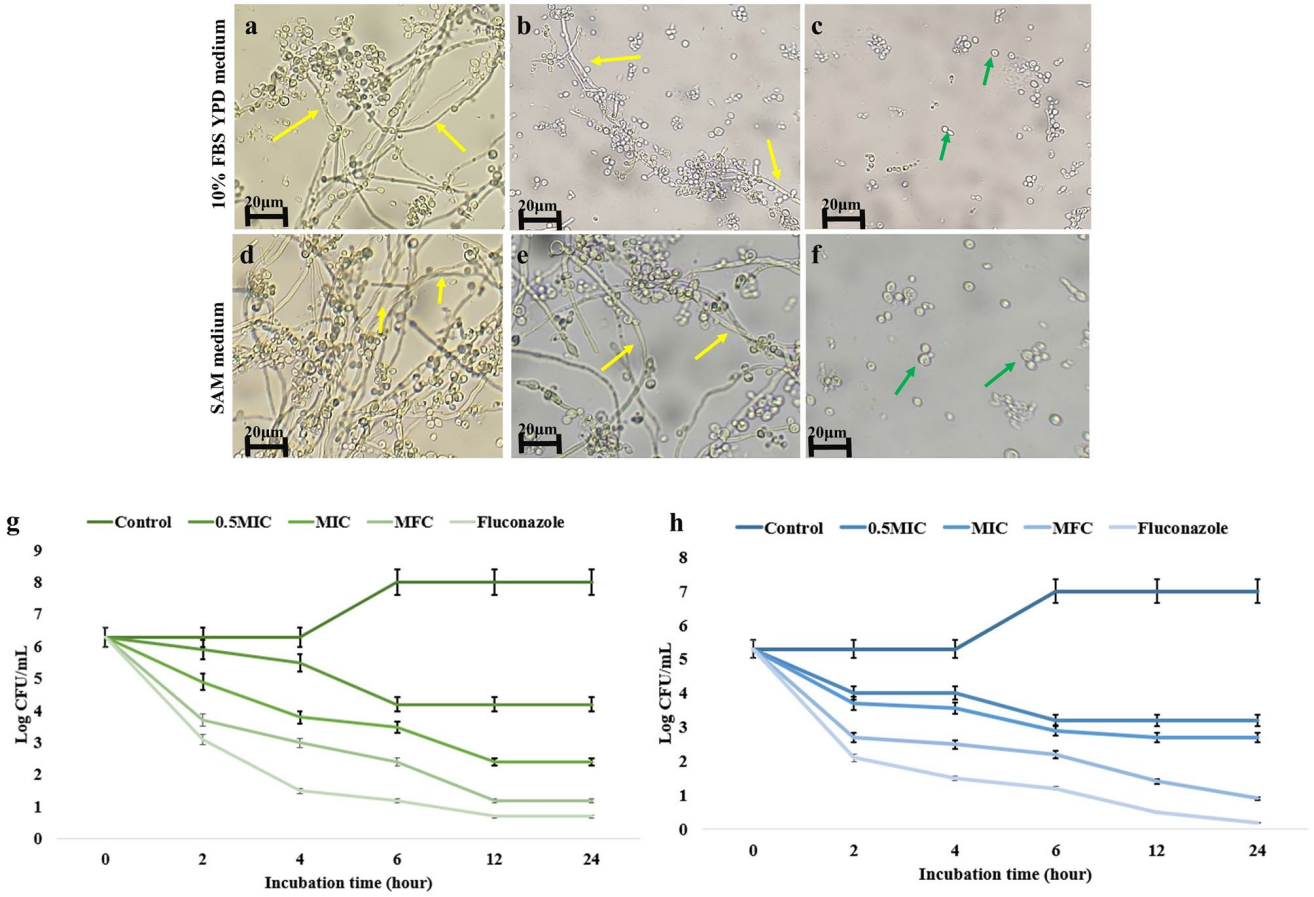
Effect of TML3 metabolites on filament development of *C. albicans* on two different filament-inducing mediums (**a–c** YPD medium with serum shift and **d–f** SAM medium) (**a** & **d**) filament development in control (not-treated with fungal metabolites) condition, (**b** & **e**) Moderate inhibition of filament development due to treatment with MIC of TML3 metabolites, (**c** & **f**) Complete inhibition of filament formation at MFC dose, Time-killing kinetics of (**g**) *C. albicans* and (**h**) *C. krusei* upon treatment with TML3 fungal metabolites exhibiting candidacidal nature of endophytic metabolites.

The fungal metabolites of TML3 inhibited the yeast cell-to-hyphal transition of *Candida albicans*. The cells were found to be arrested in only cellular form when treated with MFC doses, and no emergence of hyphae was observed (Fig. 3 a-f). Meanwhile, massive hyphal development was observed in the control set. Restriction of hyphal development strongly indicates that fungal metabolites suppress the virulence factors of the *C. albicans* pathogen.

#### Time Kill Kinetics of *C. albicans* upon treatment with fungal metabolites

TML3 metabolites were found to be fungicidal as they showed > 3log CFU reduction towards pathogenic candida cells. MFC range is the most effective candidacidal as it shows an 87% reduction of growth of pathogens compared to the control (Fig. 3 g and h). Metabolites act in both a time-dependent and dose-dependent manner. The more time (24 h for both the endophytic fungal metabolites) it gets to interact with the pathogenic cell, the more influential the results are, as the growth of pathogenic cells is inhibited drastically.

#### Endophytic metabolites block biofilm formation

**Figure 4:**
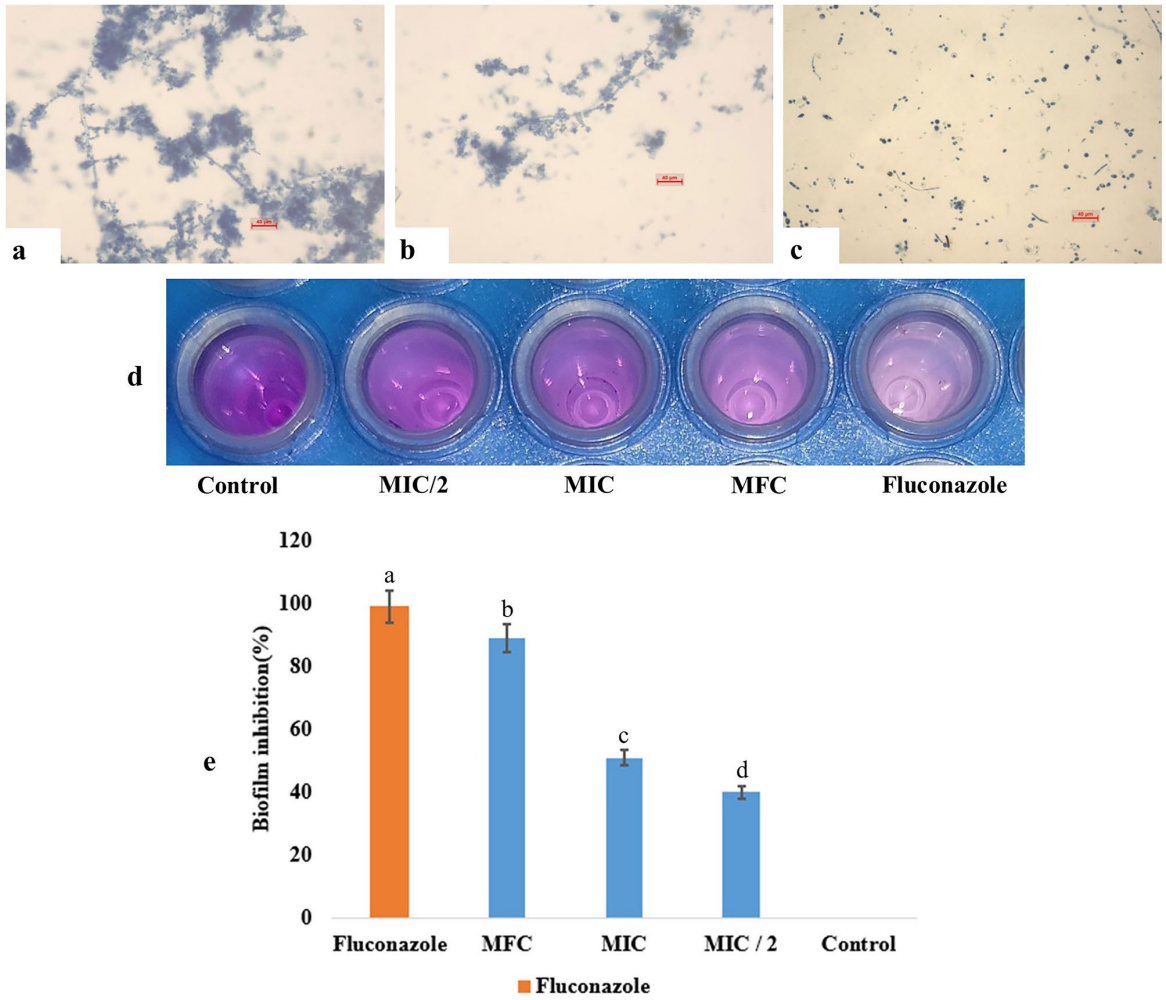
TML3 metabolites inhibit biofilm formation in *C. albicans* cells, (**a**) Biofilm formation at control condition (lacks endophytic metabolites) (**b**) and (**c**) biofilm inhibition at MIC and MFC doses. (**d**) Crystal violet-stained polystyrene cell culture plate after biofilm development in control and treated (MIC/2, MIC, MFC) conditions (**e**) Optical densities of crystal violet dye attached to the biofilm-forming cells. Values are the means ± standard error (SE) of the three replicates. The different letters indicate that there is a valid statistical difference between the treated and the control set (analysed by Tukey’s Multiple Comparison tests at At, P < 0.05 level).

Biofilm formation is a significant mode of spreading infections and antibiotic resistance in microbial pathogens. In this study, *C. albicans* was treated with TML3 metabolites, with 97.79 ± 1.34% biofilm inhibition, respectively (Fig. 4 a-e).

#### Bioactive metabolites of endophyte act synergistically with commercial antibiotics

The checkerboard-based microdilution assay revealed that TML3 fungal metabolite at a concentration of 60 µg mL^−1^ exhibited anti-candida action synergistically with antifungal fluconazole (4 µg mL^−1^). Fluconazole and TML3 metabolites were required less when applied together than in their individual applications. The FIC (Fractional inhibitory concentration) was found to be 0.494 (< 0.5), which indicates that at the above-mentioned concentrations, the interaction between the fungal metabolite and antibiotic is synergistic.

#### Endophytic metabolites hamper ergosterol synthesis

**Figure 5:**
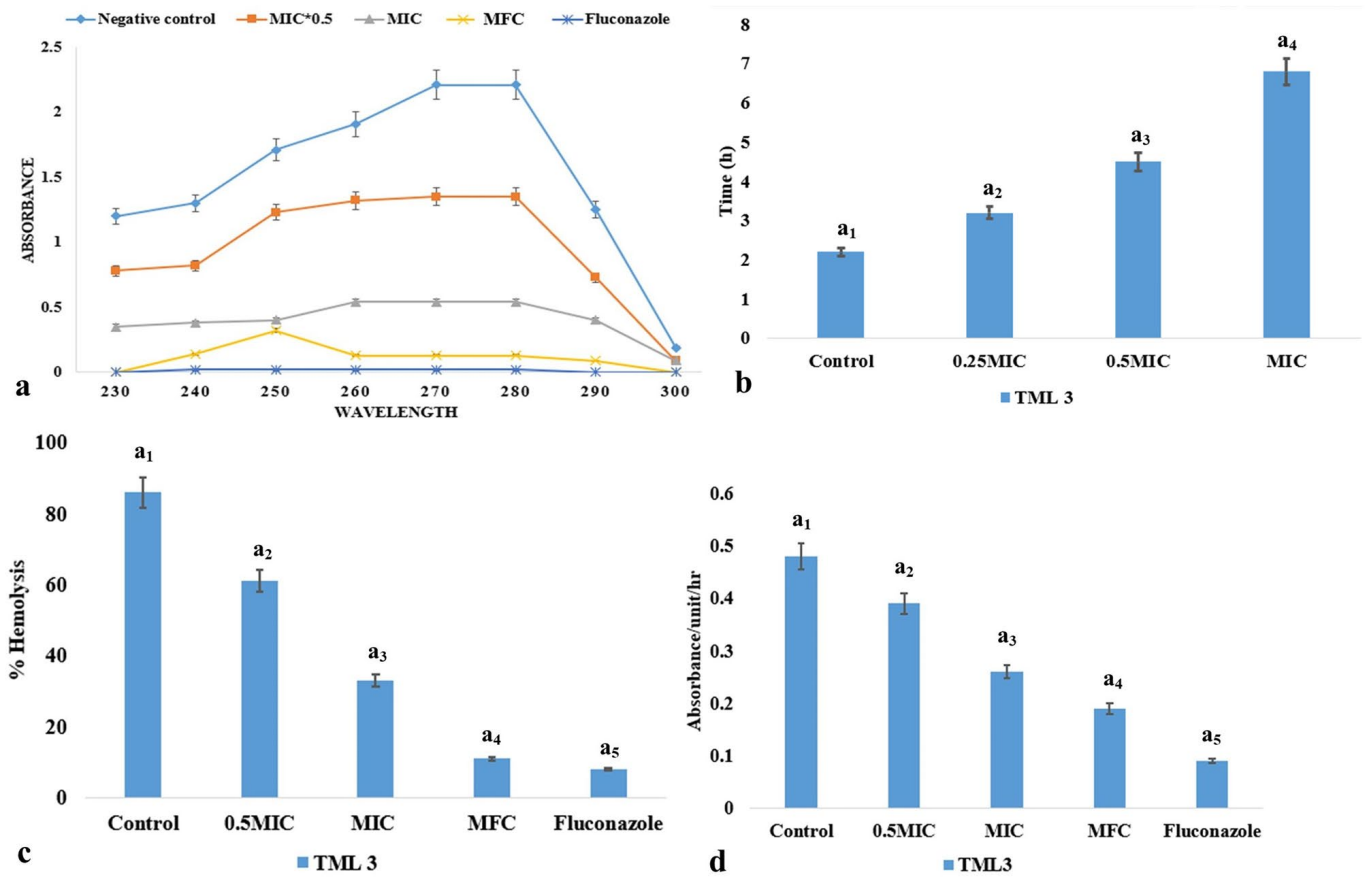
(**a**) Effect of endophytic metabolites on a- ergosterol profiles (**b**) recovery profile (post-antibiotic effect), (**c**) inhibition of hemolysis, and (**d**) Sap activity of pathogenic *C. albicans* cells. Values are the means ± standard error (SE) of the three replicates. The different letter indicates that there is a valid statistical difference between the treated and control sets (by Tukey’s Multiple comparison test) at p value < 0.005.

Inhibition of ergosterol synthesis in candida cells is an effective method to restrict the growth of the pathogen. The fungal metabolites reduced the ergosterol contents of the cells by blocking the ergosterol synthesis pathway. There was an inhibition of 26.6%, 67.4%, and 92.33% ergosterol contents when the pathogens were treated with 0.5 MIC, MIC, and MFC of TML3, respectively (Fig. 5a).

#### Post antibiotic effect

After a continuous pre-treatment of *C. albicans* cells with MIC of TML3 metabolites, the cells took 6.8 h to achieve a log growth of 1. In the control set, the cells took 2.2 h to achieve the same growth (Fig. 5b).

#### Anti-hemolytic action of endophytic metabolites

The endophytic fungal metabolites reduced the *Candida albicans*-induced hemolysis in murine cells. TML3 metabolites at MFC doses inhibited the hemolysis to 11% from 87% compared to the control (Fig. 5c).

#### Effect on secreted aspartyl proteinase (Sap) activity

TML3 metabolites maximally inhibited Sap (secreted aspartyl protein) activity and the MFC dose was the most effective (Fig. 5d). Metabolites cause a 60.41% inhibition in secreted aspartyl protein. The positive control fluconazole exhibited 81.25% inhibition.

### Characterisation of endophytic fungal metabolites

#### Characterisation of the cell-free culture extracts of TML3

**Figure 6:**
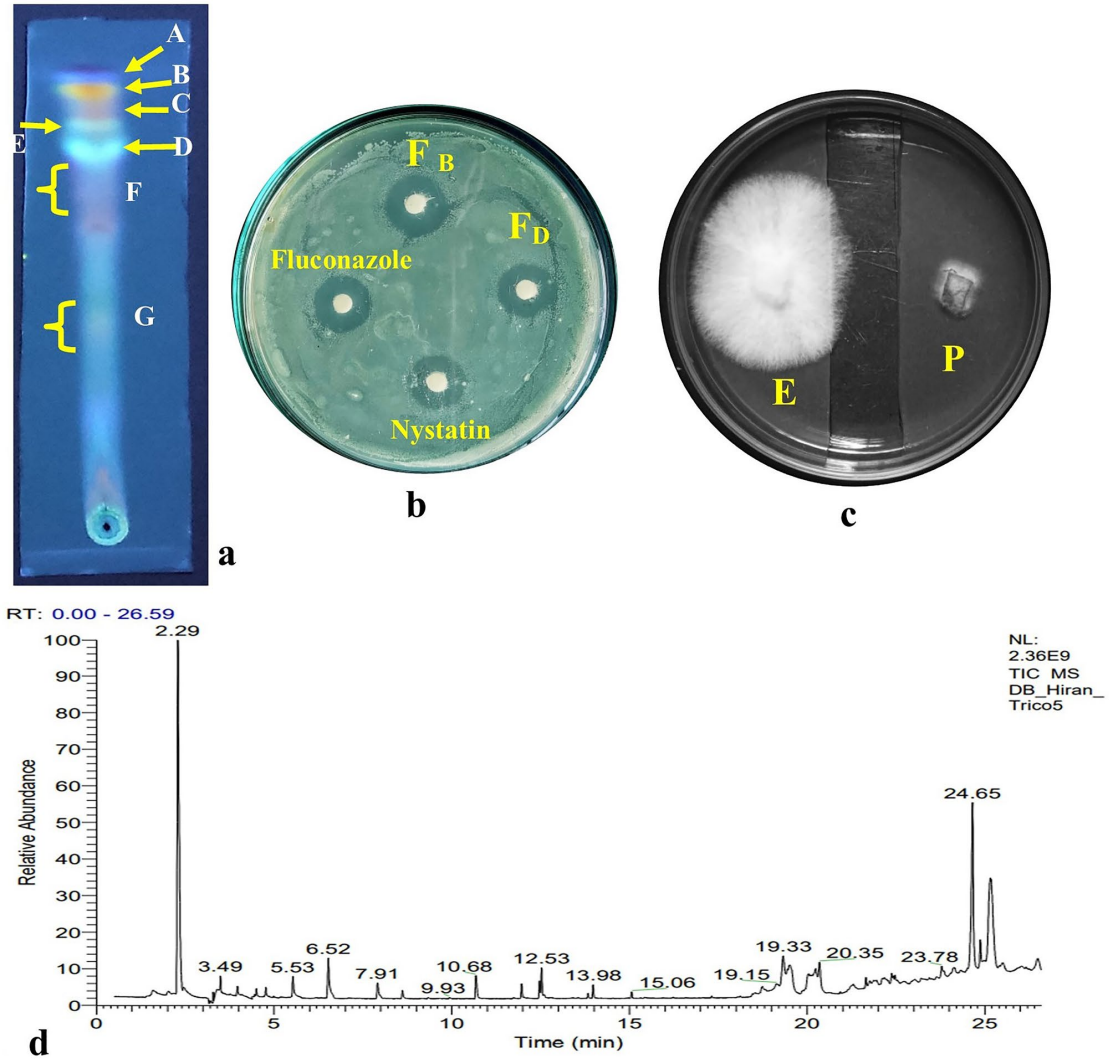
(**a**) Thin-layer chromatographic (TLC) analysis of EA fraction of TML3, (**b**) Clear zones of inhibition produced by bioactive compounds present in fractions F_B_, and F_D_ against *C. albicans*, (**c**) Volatiles of TML9 (E and P stands for endophyte and pathogen respectively) inhibits the growth of *Cercospora beticola* pathogen, (**d**) GC–MS chromatogram of the SPME analysis of the VOCs of the TML9 isolate.

EA fraction of the endophytic fungal isolate TML3 was evaluated in TLC analysis, and seven visible bands were found under UV light at 366 nm (Fig. 6a). The fractions were separately tested for their anti-candidal action, and there were two bands, B and D, with an Rf value of 0.95 and 0.81, with maximum anti-candidal action of 15 mm and 17 mm clear zone of inhibition (Fig. 6b). The fractions were dissolved in methanol, and the constituents were detected using a GC–MS instrument. Some of the detected anti-fungal metabolites are 6-pentyl-2H-pyran-2-one, 2-Nonanone, n-pentyl decanamide, 1 propanol 2-amino, 2-Amino-1-(4-methylphenyl) propane, 2-butanamine, 3-methyl (Table 3). The metabolites were non-cytotoxic towards BJ fibroblast cell lines (Supplementary Table 1) and hence can be further tested for utilization in the pharmaceutical industry.

**Table 3:** Bioactive antifungal compounds identified from band-B and band-D of EA fraction of *Colletotrichum aenigma* TML3 by GC–MS analysis.

Sl. no.	Name of the metabolites	Molecular weight (g mol^−1^)	Retention time (min)	Area (%)	Quality	Bioactivity
	Band B					
1	Dimethylsulfoxonium formylmethylide (C_4_H_8_O_2_S)	120	2.27	6.21	67.40^a^	Anti-microbial (Jemimah et al., 2015)^82^
4	6-pentyl-2H-pyran-2-one (C_10_H_14_O_2_)	166	7.89	7.41	69.87^a^	Anti-fungal against *Peronophythora litchii* and *Clarireedia jacksonii* (Yinggu et al., 2023, Liu et al., 2023)^83,84^
5	2-Nonanone (C_9_H_18_O)	142	10.67	2.14	82.19^a^	Antifungal against *Botrytis cinerea*, *Monilinia fructicola*, *M. laxa*, *Penicillium italicum*, *P. digitatum* and *P. expansum*^85^ (Calvo et al., 2020)
6	Propyne (C_3_H_4_)	40	12.52	4.15	94.95^a^	-
7	Phenethylamine (C_8_H_11_N)	121	13.97	6.21	97.26^a^	Antimicrobial (Yang et al., 2012)^86^
8	Hexa-hydrofarnesyl actone (C_18_H_36_O)	268	15.06	9.75	88.81^a^	Antimicrobial (Balogun et al., 2017)^87^
9	n-pentyl decanamide (C_15_H_31_NO)	241	19.57	31.14	73.47^a^	-
10	2,4,6-trimethyl decane (C_13_H_28_)	164	22.23	12.19	89.68^a^	Antifungal (Kumari et al., 2022)^88^
11	Tetradecane (C_14_H_30_)	198	22.39	18.19	91.23^a^	Antimicrobial (Das et al., 2023)^89^
12	4-hydroxy benzaldehyde (C_7_H_6_O_2_)	122	24.86	12.13	66.87^a^	Antimicrobial (Wang et al., 2014)^90^
	Band D					
1	5-(2-aminoethyl)-1H-imidazole-2-carbaldehyde (C_6_H_9_N_3_O)	139	3.35	29.18	78.91^a^	Antifungal (Santra and Banerjee, 2023)^91^
2	1 propanol 2-amino (C_3_H_9_NO)	75	3.79	7.31	87.76^a^	Antimicrobial (Ribeiro et al., 2023)^92^
3	Benzeneethanamine, 3-fluoro-á,5-dihydroxy-N-methyl- (C_9_H_12_FNO_2_)	185	4.1	11.17	69.18^a^	–
4	1-butanamine, N,3-dimethyl (C_6_H_15_N)	101	4.2	9.81	86.97^a^	-
5	2-Amino-1-(4-methylphenyl) propane (C_10_H_15_N)	149	4.62	5.42	77.95^a^	-
6	2-butanamine, 3-methyl (C_15_H_13_N)	87	5.21	6.57	68.91^a^	Antimicrobial (Guluma et al., 2020)^93^
7	2-methoxy-N-methylethylamine (C_4_H_11_N0)	89	6.7	4.03	78.71^a^	-
8	Pyrazole[4,5-b]imidazole, 1-formyl-3-ethyl-6-á-d-ribofuranosyl (C_12_H_16_N_4_O_5_)	296	7.31	8.50	97.16^a^	Antimicrobial (Karrouchi et al., 2020)^94^
9	Glycolonitrile (C_2_H_3_NO)	57	18.8	9.58	96.13^a^	-

Compounds detected in the control PDB liquid are not reported in the table.

^a^Compounds with a quality score ≥ 60 is identified with the spectral database of NIST.

#### Characterisation of the VOCs emitted by *C. lunata* TML9

The SPME analysis of the volatiles produced by endophytic TML9 inhibited the growth of severe phytopathogens (Fig. 6c), and the composition revealed the occurrence of volatile compounds; -trans-ocimenol, geraniol, 4-terpinyl acetate, trans carveol, trans-p-mentha-2,8-dienol, 4 caranol, 4 methyl 2 pentene, and trans verbenol (Table 4 and Fig. 6d). The standards of the emitted VOCs (three major compounds- trans ocimenol- 35.59%, Geraniol- 19.58%, 4-terpinyl acetate- 12.46%) that were available were bought from Sigma Aldrich, and the artificial atmosphere was prepared accordingly by mixing the VOCs in the appropriate amount (as emitted by the endophytic isolates and detected by the GC–MS-SPME analysis). The volatiles were also found to be non-cytotoxic towards BJ fibroblast cell lines and hence can be considered safe for the application of human consumable fruits (Supplementary Table 1).

**Table 4:** Bioactive antifungal volatiles produced by a 6-day-old endophytic fungi *Curvularia lunata* TML9 identified by GC–MS-SPME analysis.

Sl. no.	Name of the metabolites	Molecular weight (g mol^−1^)	Retention time (min)	Area (%)	Quality	Bioactivity
1	Trans-ocimenol (C_10_H_18_O)	154	2.29	35.59	93.70^a^	Antifungal (Santra and Banerjee, 2023)^91^
2	Trans carveol (C_10_H_16_O)	152	3.49	2.84	81.21^a^	Antimicrobial (Tahri et al., 2022)^95^
3	Trans-p-Mentha-2,8-dienol (C_10_H_16_O)	152	5.53	2.84	91.76^a^	Antimicrobial (Spencer et al., 2020)^96^
4	Trans-chrysanthenol (C_10_H_16_O)	152	6.52	5.33	88.12^a^	Insecticidal (Polatoğlu et al., 2018)^97^
5	Trifluoroacetyl-isopulegol (C_12_H_17_F_3_O_2_)	250	7.91	2.84	92.07^a^	-
6	2 butene, 2,3-dimethyl (C_6_H_12_)	84	10.68	3.56	89.59^a^	-
7	Terpinyl iso-valerate (C_18_H_26_O_2_)	238	12.53	4.27	91.65^a^	-
8	2,5-dimethyl-3-vinyl-4-hexen-2-ol (C_1o_H_18_O)	154	13.98	1.77	79.14^a^	-
9	4 caranol (C_10_H_16_O)	154	19.33	3.56	93.35^a^	Antimicrobial (Dalli et al., 2021)^98^
10	4-methyl-2-pentene (C_6_H_12_)	84	20.35	3.56	88.46^a^	Antimicrobial (Szkudlarek et al., 2018)^99^
11	Trans-verbenol (C_10_H_16_O)	152	23.78	1.77	88.64^a^	Insect repelling activity (Petrović et al., 2022)^100^
12	Geraniol (C_7_H_6_O_2_)	122	24.65	19.58	96.73^a^	Antimicrobial (Lira et al., 2020)^101^
13	4-terpinyl acetate (C_12_H_20_O_2_)	196	25.10	12.46	91.21^a^	-

Compounds detected in the control PDB liquid are not reported in the table.

^a^Compounds with a quality score ≥ 60 is identified with the spectral database of NIST.

#### IC_50_ value of the artificial VOC mixture

The artificial atmosphere of volatiles was prepared to mimic the VOCs of TML9, elaborated on in the last section. The antifungal activity of the synthetic mixture was evaluated against the nine pathogens discussed previously. The IC_50_ value of that artificial mixture against nine post-harvest pathogens ranged between 21.3 µL 50 mL^−1^ and 69.6 µL 50 mL^−1^ (Table 1). All the fungal pathogens were inhibited entirely at an A-VOC exposure of 150 µL 50 mL^−1^ for 144–168 h (6–7 days). The effectivity of the artificial atmosphere in antagonising fungal growth varied from pathogen to pathogen, and the volatiles acted in a highly selective and targeted manner. *P. ultimum*, *P. digitatum*, *A. alternata*, and *B. cinerea* were 50% inhibited even at a small dose of VOCs 21.3, 24.5, 27.8, 29.5 µL 50 mL^−1^ respectively, but for *C. beticola*, *A. fumigatus*, *C. ulmi*, *R. solani*, and *G. candidum* a higher dose 32, 38.6, 47.8, 53.7, 69.6 µL 50 mL^−1^ respectively is required to achieve the same inhibition. It indicates that pathogens express different patterns of sensitivity/resistance to the VOCs. *G. candidum* was resistant to fungus-emitted VOCs but was sensitive to A-VOCs.

**Figure 7:**
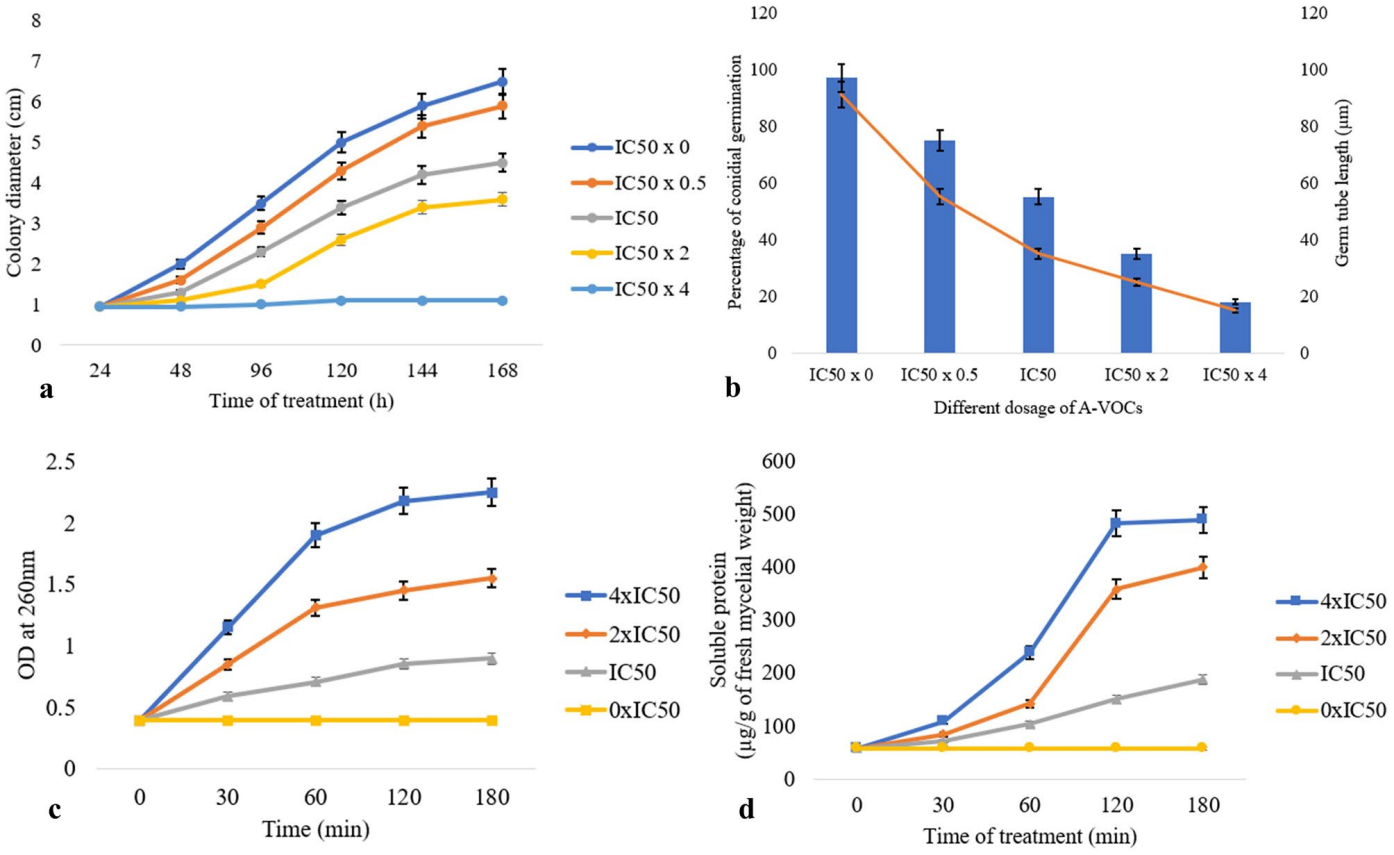
(**a**) Effect of artificial volatiles on the mycelia growth i.e., colony diameter of *P. digitatum* at different time intervals, (**b**) Inhibition of conidial germination and shortening of germ tube length of *P. digitatum* as a result of A-VOC treatment, (**c**) Effect of different concentrations of artificial volatiles on leakage of c- intracellular components detected at OD_260nm_ and (**d**) soluble proteins from mycelial cells of *P. digitatum*. Values on the graphs are the means ± standard error (SE) of the three replicates. Tukey’s multiple comparison test was performed and there was a significant statistical difference between the control and treated sets at different concentrations of volatiles and at different incubation periods (P < 0.05).

Growth kinetics in terms of inhibition of the colony diameter i.e., radial growth of the test pathogen-*P. digitatum* with an IC_50_ value of 24.5 µL was illustrated in Fig. 7a. There was a maximum inhibition of fungal growth at 2 × IC_50_ of A-VOC treatment, and the growth altogether ceased at 4 × IC_50_ dosage after 48 h of treatment.

#### A-VOCs cause leakage of macromolecules from *P. digitatum* hyphae

A-VOCs inhibited the conidial germination and germ tube elongation of *P. digitatum* in a concentration-dependent manner (Fig. 7b). At a dosage of 49 µL (i.e., 2 × IC_50_) and 198 µL (i.e., 4 × IC_50_), 18.6% and 2% conidia were able to germinate, respectively, with minute germ tube length (Supplementary Fig. 4a, b). In the untreated control set, > 96% of conidial germination was located with long germ tubes.

The leakage of UV active compounds (at OD_260nm_) from the mycelium of *P. digitatum* treated with A-VOC gradually increased with treatment time and the concentration of A-VOCs compared to the control. There was a maximum leakage of intracellular compounds from the pathogen after 120 min of treatment at 4 × IC_50_ dosage (Fig. 7c). In all three treatment concentrations of IC_50_, 2 × IC_50_, and 4 × IC_50_, the value of an optical density at 260 nm in the 120-min test group was considerably higher than that of the 0–60-min test group (P > 0.05). Therefore, it may be concluded that the release of cell components increased over the treatment, reaching its peak at 120 min. The protein leakage profile of the A-VOC-treated *P. digitatum* is illustrated in Fig. 7d. The leakage of soluble proteins into the extracellular environment increased concurrently with the incubation time, much as the leakage of intracellular components at OD_260_. *P. digitatum* cells leak 151, 349, and 483 soluble proteins (mg/g fresh mycelium) in response to treatments with IC_50_, 2 × IC_50_, and 4 × IC_50_ dosages respectively. Only 59 mg of protein leakage was found in the control set after 120 min of incubation. VOCs also inhibit the activity of specific enzymes involved in central carbohydrate metabolism (Supplementary Fig. 5) and disrupt the cells of the treated pathogens, as seen under scanning electron microscopy (Supplementary Fig. 6).

#### Optimization of VOC production

Endophytic TML9 was cultured on different solid media (with varying nitrogen and carbon sources) to check the optimum production of volatiles with a characteristic odour. The occurrence of a maximum amount of trans-ocimenol and geraniol emits a higher degree of sweet citrus and rose-like fragrance. The emission of a higher or lower degree of the strong fragrance was tested by an olfactory score of 0–5. The most appropriate media with the maximum olfactory score was selected (Supplementary Table 2). This media composition also reported a maximum amount of biomass production and anti-fungal activity. Further, for applying this potent VOC-emitting isolate TML9 as a post-harvest pathogen control agent, it was grown on cheap agri-waste wheat husk, and the formulation was named T9-WH. Besides six cheap agri wastes tried for this formulation, TML9 inoculated in wheat husk yields maximum volatile emission and anti-fungal activity (Supplementary Fig. 7).

#### T9-WH controls green mold infection in oranges

*Penicillium digitatum*-infected (Group-II) oranges were found to be highly damaged due to the invasion of green mold. Fruits started to rot quickly, and the pathogen occupied the internal tissues of the tested fruits, green-coloured thick mat of mold colonised all over the fruit, starting from the sights of pathogen inoculation. The fruits started to decay after 5th day of inoculation of the green mold. All six fruits lost their natural texture due to the fungal attack, and the healthy orange color became pale first, and then a population of green spores occupied the fruits (Supplementary Fig. 8a). The fruits inoculated with the fungal pathogen and then exposed to T-WH volatiles (group-III) remained healthy throughout the experiment (Supplementary Fig. 8b). The pathogenic invasion was also located in group III, but it was restricted to the site of pathogen inoculation, and there was no immediate tissue damage or massive green mold growth even after twelve to fifteen days of inoculation. T-WH fumigated oranges were microscopically observed, and no such massive necrotic lesions were reported, whereas in the control set, severe necrotic masses were located. So, the endophyte TML9 formulated here with wheat husk as T-WH emits novel anti-fungal VOCs and can be commercially exploited for post-harvest disease management of oranges. The viability of green mold after T-WH exposure was evaluated by re-isolating the inoculated pathogen from the oranges. There was a 20 ± 2% isolation frequency of *P. digitatum* from the infected oranges. The findings show that 80% of pathogenic development in vivo was inhibited following VOC treatment. The VOC-treated fruits were found to retain their qualities in terms of total sugar content (%), pH, ascorbic acid content (mg/100 g-fresh weight), and percentage of soluble solids (Supplementary Table 3).

## Discussion

Microbial infection is one of the prime healthcare concerns globally. Deaths due to antimicrobial resistance (AMR) and the emergence of multidrug-resistant strains (MDR) have increased drastically over the past few decades^26^. The irrational applications of antimicrobial drugs and subclinical infections have worsened the situation. The solution can only be found from novel natural sources in these circumstances. Besides plant metabolites, nowadays, scientists are shifting their search to a new group of microbial symbionts called endophytes. These are considered the most dependable source of bioactive compounds with multifaceted utility and antimicrobials from endophytes of medicinal plants are top of that list^27^. The plant *T. majus* possesses a unique metabolome with multidomain bio-utility^20^. Keeping these facts in view we selected the plant for our study. The plant was collected from the Darjeeling Himalayan region (around Lloyd Botanical Garden) and was evaluated for its antifungal-producing endophytes. Till now, there has been a very limited number of discoveries on the endophytic microflora of this plant, so we explored the plant for its untapped hidden endophytic treasure. In total, fourteen endophytic fungi were isolated from the explants, and *C. aenigma* TML3 was found to be the most frequent colonizer among the isolates and was the most potent anti-candida producer. *C. lunata* TML9 synthesizes volatile antifungals and restricts the growth of phytopathogens. Volatile and non-volatile compounds were analysed using the GC–MS-NIST library and written as such (Tables 3, 4). The EA fraction of TML3 was evaluated for their mode of action against human pathogenic *C. albicans* (MTCC-4748). TML3 exhibited minimum fungicidal concentration ranging from 200–600 µg mL^−1^ against four candida pathogens. Endophytic metabolites restrict the candida growth by altering the cell membrane permeability, which leads to lethal leakage of biologically valuable macromolecules, i.e., DNA, protein, K^+^ ions, other UV active housekeeping enzymes, cytoplasmic β-galactosidase (necessary for pre-glycolytic pathways) needed for normal cellular growth and metabolism. The entry of crystal violet stain in a higher percentage is also solid proof of damage to the candida cell membrane^28^. Hemolytic action and Sap (secreted aspartyl proteinase) activity of *C. albicans* were also blocked by treatment with fungal metabolites. So, the endophytic metabolites neutralize the toxins responsible for the hemolytic activity of the pathogen^29^. The proteinase activity of the pathogen is directly proportional to the invasive potentialities, and tissue-damaging capacities, and a block of that proteinase activity ensures cellular protection from pathogenic invasions^30,31^. The outcomes of the killing kinetics experiment led to the fact that the metabolites are candidacidal and drastically reduce the growth of the pathogen. Chatterjee et al. (2022) reported similar action of metabolites produced by endophytic *Alternaria tenuissima* PE2, isolated from *Psidium guajava*^32^. The metabolites act at both time and in a concentration-dependent manner. Exposure of pathogenic cells for 24 h with MFC doses exhibits a maximum growth reduction of 87% (a sharp increase of 25.71% from 12 h treatment at MIC dose). Disruption of the central carbohydrate mechanism due to blockage of the three most physiologically active enzymes is one of the prime causes of anticandidal activity of the metabolites. Blocking valuable enzymes is a popular mode of action for endophyte-originated metabolites with antimicrobial potentialities^33,34^.

Endophytic metabolites were effective against four different species of *Candida*, both albicans and non-albicans. However, the albicans species were found to be the most sensitive in response to endophytic metabolites, so this strain was selected for further studies. The endophyte-produced metabolites are non-toxic to BJ fibroblast cells; thus, their potential to be exploited for developing novel anti-candida drugs is high. Also, the anti-Candida metabolites were effective in vivo conditions. They blocked the expression of the following virulent-causing genes, *Sap1*, *Erg3*, and *Erg11*, which were involved in the pathogenic action of *C. albicans*. *Candida albicans* is a serious pathogen causing fatal infections in female or male urinogenital tracts, candidemia, and the dreadful condition of sepsis. Patients facing immunosuppressive treatments i.e., post-organ or bone marrow transplantation, under-going chemotherapy, kidney failure, and persons infected with AIDS face high morbidity due to *C. albicans* infections. The situation is worsening day by day and the available anti-candida drugs are developing fast resistance. The two most virulent features of candidiasis are the transition of yeast to hyphal form and the rapid production of thick, resistant, hardly permeable biofilms^35^. Till now, there has been the discovery of a variety of drugs that target superficial as well as invasive candida infections but all of them are developing quick resistance and thus making the situation extremely tough to control. Endophytic metabolites restrict the transition of yeast to hyphal form, thus controlling the invasion and dissemination of the virulence factor to the mammalian host cells. Active principles of endophytic *Alternaria tenuissima* isolated from leaf tissues of *Ocimum tenuiflorum* restrict the candida growth following a similar mode of action; blocking the biofilm and hyphae formation^9^. Chowdhury et al. (2022) reported the anti-biofilm activity of *Pestalotiopsis microspora* isolated from *Dillenia pentagyna*^36^. Over-administration of antimicrobial agents (i.e., antibiotic or antifungal) exhibits dreadful consequences in human bodies other than the development of antimicrobial resistance; issues related to gastrointestinal dysbiosis, psychiatric and neurological imbalances, and hepatic disorders are often reported. That’s why, instead of using a single drug in high concentration, the combined effect of two synergistic agents may improve the overall outcome. In this case, even a lower dose of fluconazole (less than MIC dose), when applied along with TML3 metabolites, exhibits similar or superior anti-candida activity. Fluconazole blocks the ergosterol metabolism of the candida pathogens and interferes with cell membrane formation. The co-application of TML3 metabolites and fluconazole may improve antifungal medication's overall impact. The metabolites interfere with the ergosterol biosynthesis of the candida cells. A sharp decrease in ergosterol contents occurs due to a reduction in its synthesis and an alteration in membrane integrity^37,38^. The reduction in ergosterol content is usually facilitated by blocking (down-regulation) the transcription factors *Erg3* and *Erg11*^39^. Endophytic metabolites of *Rosa damascena* Mill. exhibits anticandidal activity by blocking the ergosterol synthesis^40^.

The endophytes are effective against human pathogenic fungi but also efficiently tackle phytopathogenic fungi. The VOCs produced by a 6-day-old endophytic *Curvularia lunata* TML9 restrict the growth of the phytopathogens up to 89% except for *Geotrichum candidum*. In the present investigation, VOCs have eliminated several severe phytopathogens, including *Rhizoctonia solani*, *Alternaria alternata*, *Botrytis cinerea*, *Cercospora beticola*, *Penicillium digitatum*, *Ceratocystis ulmi*, and *Pythium ultimum*, from the different classes- Ascomycota, Basidiomycota, Oomycota, and Deuteromycota^41,42^. These fungi are responsible for a range of illnesses, including leaf spot, soft rot, fruit rot, necrotrophic lesions, damping off, and root rot in a variety of income crops- wine grapes, rapeseed, wheat, corn, fir, soybean, potato, tomato, tangerine, strawberry, tobacco, etc. from diverse families- Solanaceae, Brassicaceae, Poaceae, Rutaceae, Rosaceae, Fabaceae etc. leading to devastating economic loss^43–46^. Besides phytopathogens, one mammalian pathogen *Aspergillus fumigatus*, a causal agent of aspergillosis in immunocompromised patients, was also killed by VOCs^47^. In previous reports, different VOCs- 1-Propanol, 2-methyl-, 2-propanone, sabinene, 1-Butanol, 3-methyl, gymnomitrene, beta-acoradiene, dodecanoic acid, hexadecenoic acid, 1-Butanol, 3-methyl-, acetate, propanoic acid-2-methyl, α-Humulene, β-Selinene, aromadendrene, α-amorphene, β-himachalene, cadinene emitted from endophytic fungi- *Phoma* sp., *Phomopsis* sp., *Muscodoralbus*, *M. brasiliensis*, *M. sutura*, *M. ghoomensis*, *M. cinamommi*, *M. camphora*, *Nodulisporium* sp., have found to restrict a variety of pathogens^48–52^. These VOCs were tested for their cytotoxicity towards BJ fibroblast cell lines. They were found to be non-cytotoxic, increasing the chances of being used to treat fungal infections in consumable fruits.

To evaluate the antifungal activity of the emitted compounds more effectively, an artificial mixture was prepared mimicking the ratios of significant VOCs (trans-ocimenol-35.59% and geraniol-19.58%) as released by TML9. It was found that the VOCs are biologically selective and inhibit all eight pathogens to different degrees; thus, this could be used as a potent myco-fumigator and can restrict phytopathogen growth. Applying VOCs reduced the green mold infections in *Penicillium digitatum*-infected sweet oranges (*Citrus sinensis*). As VOCs are potent biocontrol components synthesised from endophytic fungi, they must have formulations for their prolonged and extensive use in post-harvest facilities. The VOCs maintain the critical quality parameters of the treated fruits and thus can be exploited for broad-agricultural purposes. VOCs of TML9 effectively block the action of the enzymes involved in central carbohydrate metabolism and cause leakage of major macromolecules and cations from the pathogenic cells. The rugged appearance of the cell membrane of the TML9-VOC treated hyphae confirms the disruption of the pathogenic cells. A cheap agri-waste- wheat husk (T9-WH), packed in a cotton bag, was used as the carrier agent of the VOC-emitting endophytic fungi TML9. Yeh et al. (2020) formulated PDL-005 products using bagasse as the media and reported maximum anti-*P. digitatum* activity of the VOCs of endophyte *Nodulisporium* sp.^53^. VOCs are useful in post-harvest disease management, protection of standing crops, or even in improving the shelf life of cash crops^33^. In most cases, VOCs interact with the cell wall and cell membrane of phytopathogens, decreasing their integrity and increasing the permeability of their plasma membrane, leading to leakage of soluble proteins and UV (260 nm) active substances^54^. Additionally, an increase in reactive oxygen species (ROS) and a loss of the distinctive colour have also been linked to antifungal action^55–57^. Diffusing through the cell membrane they operate as transcriptional regulators and modulate the expression of genes involved in maintaining redox balance and cell wall integrity, i.e., chitin synthase, chitinase, glycerol-3-phosphate dehydrogenase, glutathione peroxidase, glutaredoxin, thioredoxin, endo-1,3(4)-beta-glucanase, cell wall integrity and stress response component, cell division control protein 6, and programmed cell death protein 5, etc.^25^. Restriction of phytopathogens and human pathogens by the action of endophytic metabolites is a popular method of tackling fungi-related parasitism/infection^58,59^. This can be a potential approach toward sustainable agricultural practices. In a word endophytes of *T. majus* represent a plethora of volatile and non-volatile bioactive compounds with candidacidal/fungicidal activity.

## Materials and methods

### Isolation of endophytic fungi

Fungal endophytes were isolated from symptomless tissues (leaves and stem) of *T. majus*. Tissues were surface sterilized under running tap water to remove debris^60^. In the following steps, samples were rinsed with 70% ethanol for the 30 s under aseptic conditions, and the leaves and the stem were treated with 0.1% sodium hypochlorite for one minute. After each sequential rinsing step, plant parts were cleaned with autoclaved distilled water 2–3 times. Washed samples were dried on filter paper. Small 5 mm × 5 mm explants were excised from these sterilized samples. They were plated on a water agar medium previously supplemented with antibiotic streptomycin to avoid bacterial contamination and bacterial endophytes. The plates were incubated at 28 ± 2 °C for 5–8 days. Explants were regularly monitored for any fungal growth, and any emerging fungal hyphal tips were immediately transferred to PDA (Potato Dextrose Agar) medium^13^.

### Screening of fungal metabolites for anti-fungal activity

#### Evaluation of crude extracts for anti-candidal activity

Fungal isolates were grown on 100 mL of potato dextrose broth (PDB) in a 250 mL Erlenmeyer flask for 5–8 days at 28 ± 2 °C under 120 rpm agitation. Cell-free culture extracts were obtained by centrifugation at 5000 rpm for 10–12 min. Crude cell-free culture extracts were evaluated for their anti-candidal action against *Candida albicans* (MTCC 4748), *C. glabrata* (ATCC 2001), *C. krusei* (ATCC 6258), *C. tropicalis* (MTCC 184) by disk diffusion method. In brief, 5 mm diameter sterile disks were soaked with 50 μL of culture filtrate and were placed at the PDA (Potato Dextrose Agar, HiMedia, India) plates inoculated with test microorganisms and incubated at 28 ± 2 °C. After 24 h, inhibition zones (in mm) were observed, and diameters were measured. Fluconazole (HiMedia) was used as a standard antibiotic. In vivo analysis of anti- *Candida albicans* activity of TML3 metabolites and In vitro gene expression (Supplementary Table 4) of *C. albicans* upon exposure to TML3 metabolites was performed following the standard procedures discussed in detail in the supplementary section.

#### Evaluation of endophytic fungal isolates for production of anti-fungal volatile organic compounds (VOCs)

The isolates were further checked for their antifungal VOCs (Volatile organic compounds) production against selected pathogens *Rhizoctonia solani* (MTCC-4634), *Alternaria alternata* (MTCC-3793), *Geotrichum candidum* (MTCC-3993), *Botrytis cinerea* (MTCC-8659), *Cercospora beticola* (ATCC-12825), *Aspergillus fumigatus* (MTCC-3785), *Ceratocystis ulmi* (ATCC-32437), *Pythium ultimum* (ATCC-200006), *Penicillium digitatum* (ATCC-34644) following a bioassay method proposed by^51^. Antifungal VOC production was checked at different time intervals (after four five, six, and seven days). A 1-cm wide agar strip was cut off from the center of a standard Petri plate (100 × 15 mm) of PDA, forming two halves of agar. Endophytic fungi were inoculated onto one side of the plate (half-moon agar) and incubated at 24 °C for 4–8 days for emission of VOCs. Test pathogenic fungi were inoculated on the other half-moon agar side of the Petri plate from the fourth until the eighth day. Petri plates were then wrapped two or three times with parafilm and incubated at 24 °C for 48–96 h. The growth of filamentous fungi was quantitatively assessed based on multiple growth measurement criteria relative to control. Tests were conducted in triplicate.

#### Identification of potent fungal endophytes and phylogenetic analysis

The endophytic fungi were identified based on their microscopic and macroscopic morphology using the standard literature. The microphotographs of the sterile and spore-bearing structures of the fungal hyphae were taken using a light compound microscope (Primo Star, Zeiss, Germany). The most potent endophytic fungal isolates with the broad spectrum anti-fungal activity and VOC production ability were sterile even after growing them in carnated leaf agar (CLA) medium. They were identified following the molecular techniques. DNA of the selected endophytic fungi TML3 and TML9 were isolated and amplified through PCR using ITS1 (5′TCCGTAGGTGAACCTGCGG 3′) and ITS4 (5′TCCTCCGCTTATTGATATGC3′) universal primers^61^. These primers amplify ITS regions by targeting conserved 18S and 28S rDNA regions of rDNA^58^. Amplified segments were sequenced from Bioserve Biotechnologies, Hyderabad, India. Sequences were analysed using the BLAST search tool (NCBI) and were submitted to the GenBank database. Next, the sequences were aligned and a phylogenetic tree was constructed using Megaversion-10 software. Neighbour-joining method was used for the evaluation of the phylogenetic tree^58^.

### Evaluation of mode of action of endophytic fungal metabolites

#### Determination of minimum fungicidal concentration (MFC)

Anticandidal activities of EA fraction of TML3 cell-free culture extract were determined against *C. albicans*, *C. tropicalis*, *C. glabrata*, *C. krusei* by disk diffusion technique described earlier^63^. EA fractions were dissolved in DMSO in different concentrations, and 20 µg mL^−1^ to 5 mg mL^−1^ of culture extract were prepared according to the broth microdilution method for the determination of minimum fungicidal concentrations (MFC) in a 96-well round bottom microtiter plate (Merck, Germany) following the guidelines of Clinical and Laboratory Standards International (CLSI, 2017). 100 µL of the fungal extract with varying concentrations (0.195 µg mL^−1^ to 3.2 mg mL^−1^) were added to 100 µL of Sabouraud dextrose broth (SDB) (1.5 × 10^8^ CFU mL^−1^ prepared according to the 0.5 McFarland turbidity standard) in wells. Antifungal fluconazole and SDB (containing Candida only) were selected as positive and negative controls, respectively. The plate was incubated at 37 °C for 24 h. The optical density in each well was quantified using a microplate reader (ROBONIK read well Touch ELISA Plate Analyser, India).

### The activity of fungal metabolites on candida membrane permeability

#### Candida membrane integrity assay

The effect of the endophytic fungal extract (TML3) on candida cell membrane integrity was studied by evaluating the discharge of intracellular macromolecules i.e., DNA, protein, and K^+^ in the extracellular environment following the standard procedures. *Candida albicans* were grown (OD620 nm = 0.5) in 250 mL SD broth, and pathogenic cells were harvested by centrifuging the culture broth for 10 min at 6,000 rpm. The cell pellets were taken and washed two times with a 50-mM Na-P buffer (pH, 7). The cell pellets were resuspended in 1 mL of the same 50-mM Na-P buffer. Then, the pellets were treated with EA extract of TML3 at its MIC and MFC values. After 6 and 24 h of incubation, the solution was centrifuged at 10,000 rpm for 10 min to obtain the cell-free extracts. Protein and DNA concentrations in the mixture were determined following the standard methods^63,64^ using a Shimadzu UV 1800 spectrophotometer (Japan). The concentration of K^+^ ions was calculated using a flame photometer (Elico, CL-378, India), with K_2_HPO_4_ (Merck, Germany) as a standard. The set that was only treated with DMSO was considered a control.

#### Crystal violet assay for membrane permeability

The crystal violet assay evaluated the ability of the endophytic fungal metabolites to alter the membrane permeability of the pathogenic candida cell. The ability of the endophytic fungal metabolites to alter the membrane permeability of the pathogenic candida cell was evaluated by the crystal violet assay^65^. The candida cells were cultured in sabouraud dextrose broth and centrifuged at 10,000* g* for 5 min to collect the pathogenic cells as a pellet. The cell pellets were washed and suspended in a 0.5% NaCl solution. Specific concentrations (½ MIC, MIC, and 2 MIC) of the fungal metabolites (cell-free culture extracts of TML3) were added to the pathogenic cell suspension and incubated for 6 h at 37 °C. The whole suspension was centrifuged and the obtained cell pellets were resuspended in crystal violet (10 µg mL^−1^) supplemented with 0.5% NaCl and incubated for 10 min at 37 °C. The optical density of the supernatant was measured at 590 nm. The % of crystal violet uptake was calculated as (OD value of the fungal metabolite treated sample/ OD value of crystal violet solution) × 100.

#### Assay of candida inner membrane permeability and effect on the central carbohydrate metabolism

Discharge of cytoplasmic β-galactosidase, fructose-1,6-bisphosphatase, phosphofructokinase, and mitochondrial isocitrate dehydrogenase from the candida cells upon treatment with endophytic fungal metabolites (TML3) was detected following the standard procedures. In brief, for β-galactosidase action, the candida cells were cultured in SDB, and the cell pellets obtained through centrifugation were suspended in 0.5% NaCl. Then 200 µL of cell suspension was taken in a 96-well plate along with 10 µL substrate o-nitrophenyl –β-D-galactoside ONPG. They were treated with different concentrations of fungal metabolites. The release of o-nitrophenol was colorimetrically (UV–VIS, Shimadzu, 1700) evaluated at a wavelength of 415 nm^66^.

The other three physiologically valuable enzymes, FBPase (fructose-1,6-bisphosphatase), PFK (Phosphofructokinase), and ICDH (isocitrate dehydrogenase), were assayed following the methods of^65^. The candida pellets dissolved in 0.5% NaCl were mixed with the enzyme substrate and co-factors in a 96-well plate. The rate of NADP reduction was followed at 340 nm in a UV–Vis spectrophotometer (Shimadzu UV-1700). The specific activity was calculated as nanomoles of substrate consumed per min per mole protein and compared to the untreated control.

#### Filamentation and hyphal growth assays

The effect of the EA fraction of TML3 on the filament formation of *C. albicans* was evaluated following the standard methods^67,68^. Candida cells were grown overnight in YPD (yeast extract 10 g L^−1^, peptone 20 g L^−1^, dextrose 20 gL^−1^, pH 6.5) medium supplemented with fetal bovine serum (10%), and SAM (Spider agar medium- nutrient broth 10 g L^−1^, mannitol 10 g L^−1^, K_2_HPO_4_ 2 g L^−1^, agar 13.5 g L^−1^ with a pH of 7.2) medium respectively for the induction of filament. The specific concentrations of the EA extract (0.5 MIC and MIC) were added to the suspension and the yeast to the hyphal formation (in the control set- without any fungal EA extract), and also the inhibition of hyphal formation (in treated sets) was photographed by Light microscope (Primo Star, Zeiss, Germany).

#### Assay for time-kill kinetics

The killing kinetics experiment was performed following the standard guidelines^69^. Fresh cultures of *Candida albicans* were prepared separately in SDB and were suspended in sterile saline water. The culture turbidity was adjusted at 0.5 McFarland’s standard for inoculation. The suspension was diluted further to obtain an inoculum of 5 × 10^7^ CFU mL^−1^. DMSO dissolved EA extract (TML3) was prepared in different concentrations (MIC/2-half of MIC, MIC, MICx2-double of MIC) and was added to different tubes containing a suspension of *Candida albicans* separately. Five sets of candida samples, and different concentrations of fungal EA extract, were prepared. No addition of the fungal metabolites to a flask indicates the negative control. Candida cultures were harvested at different time intervals from the experimental setup, and in total, six time points (0, 2, 4, 6, 12, and 24 h) were selected for sampling following centrifugation at 37 °C at 110 rpm. The samples were serially diluted and re-cultured in the Sabouraud dextrose agar (SDA) medium and then incubated overnight at 37° C. To determine log CFU values, the plotted graph was defined as cidal or static based on the CFU reduction at various time intervals. The fungal metabolites were said to be candidacidal if they exhibited ≥ 3 log CFU (colony forming unit) reduction and candida-static if they exhibited < 3 log CFU reduction.

#### Evaluation for antibiofilm capacity

Biofilm formation of the *C. albicans* cells was tested using a 24-well polystyrene cell culture plate following the standard methods. 1 mL of SD broth was poured into each well and then inoculated with 1% fresh culture of *Candida albicans*. The EA fraction of TML3 (dissolved in 10% sterilized DMSO) was added at different concentrations to different wells. After an incubation of 48 h at 27 °C, the broth was decanted from every well and then washed with sterilized distilled water without hampering biofilm formation. Afterward, each well was dried and washed gently with sterilised water without disturbing the biofilm. Next, the wells were stained with 1 mL of 0.1% crystal violet (SRL) and kept at room temperature for 10 min. The crystal violet stain was washed, and 1 mL of 33% acetic acid (SRL) was added to each well and then kept for 30 min at room temperature, providing mild agitation to extract the bound crystal violet from bacterial cells. The acetic acid solution's optical densities (OD) were then measured at 595 nm using a UV–vis spectrophotometer. The inhibition of the biofilm was determined according to formula^71^. %Inhibition of biofilm formation = 100 − {(OD570 of sample/OD570 of control) ∗ 100}. The biofilm formation was classified at three levels: the highest one (OD570 ≥ 1), intermediate (0.1 ≤ OD570 < 1), and no formation of biofilm (OD570 < 0.1).

#### Interaction with a standard antifungal agent

The synergistic anticandidal activity of the TML3 EA fraction and clinically proven anticandidal drug fluconazole against *Candida albicans* were tested following the checkerboard method proposed by Orhan et al. (2005)^71^ with slight modifications (Prinsloo et al., 2008)^72^. The EA fraction of the endophytic fungi TML3 (0–1000 µg mL^−1^) and the most effective antifungal fluconazole (0–100 µg mL^−1^) were used at variable combinations. A 1 mL of Sabouraud dextrose (SD) broth was taken to a 1.5 mL micro-centrifuge tube and then inoculated with 1% *C. albicans* culture (OD620 nm = 0.5). TML3 EA extract and fluconazole were mixed at variable combinations. The mixture was incubated at 28 °C for 48 h. Cell pellets were drawn after centrifugation for 10 min at 6000 rpm and washed thoroughly two times in 50 mM Na-P buffer. Next, the candida cells were suspended in 1 mL of the same buffer and the OD values were measured at 620 nm to quantify the cellular amounts. Different OD values were placed on the checkerboard to calculate the ∑FIC (fractional inhibitory concentration). ∑FIC was calculated by the following formula, ∑FIC = FIC of EA fraction + FIC of fluconazole, where the FIC of the EA fraction or fluconazole = MIC of the EA fraction or fluconazole in combination/MIC of the EA fraction or fluconazole alone. The endophytic culture extract and fluconazole combination was considered antagonistic when ∑FIC > 4, synergistic when ≤ 0.5, and indifferent when > 0.5.

#### Ergosterol quantification

The ability of the endophytic fungal metabolites (TML3) to alter the candida growth in terms of hampering the ergosterol metabolism is evaluated following the methods of Khan et al. (2013)^37^. In brief, candida cells (1.5 × 10^8^ CFU mL^−1^) were suspended in SD broth, and specific concentrations of endophytic fungal metabolites (TML3 culture extract) were added and incubated for 48 h. After that, the cells were harvested, washed with sterile water, and desiccated at room temperature. Each pellet's weight was recorded and suspended in 3 mL of 25% alcoholic KOH, at 90 °C for 1 h. A solution of 3:1 n-heptane/water was used to extract the sterols and the heptane layer was retrieved after 30 min and stored at −20 °C until analyzed. A five-times dilution of the eluent in ethanol was recorded spectrophotometrically with a wavelength of 230–300 nm. The generation of four peaked curves indicated the detection of ergosterol and DHE (24(28) dehydroergosterol). The percentage of ergosterol was evaluated following the equations of Khan et al. (2013). Candida cells not treated with fungal metabolites and antifungal fluconazole were taken as control respectively.

#### Post antibiotic effect on the growth of *C. albicans*

The pathogenic cells of *C. albicans* at a CFU of 1.5 × 10^8^ mL^−1^ cells were inoculated in SDB, and the medium was supplemented with specific concentrations (0.25 MIC, 0.5 MIC, and MIC) of the fungal metabolites and incubated for 2 h. The culture was centrifuged, the cell pellets were washed in sterile SDB, and finally resuspended into 5 mL SDB. The post-antibiotic effect was measured by calculating the CFU mL^−1^ at an interval of 1 h to achieve the 1 log10 CFU mL^−1^ growth. The time required for the untreated control sample to achieve similar growth was compared with the treated one^12^.

### Effects of fungal metabolites on virulence factors of *C. albicans*

#### Haemolysis assay

Haemolytic toxicity of the pathogenic candida was evaluated using murine erythrocytes following the methods of Kaur et al. (2017). 10 mL of murine blood was centrifuged for 5 min at 2500 rpm, and pellets were cleansed using phosphate buffer saline (PBS). PBS was also used to prepare the erythrocyte suspension (3%). Different concentrations (1/2 MIC, MIC, and 2MIC) of endophytic fungal metabolites (cell-free EA extracts of TML3) were mixed with the murine erythrocyte suspension (10 µL). The supernatant was collected after centrifugation of the mixture at 2500 rpm for 5 min, and the optical density was recorded spectrophotometrically (Shimadzu UV-1700, USA) at 540 nm to detect the amount of hemoglobin released^73^. Erythrocyte suspension dissolved in sterile distilled water was considered blank.

#### Secreted aspartyl proteinase (Sap) inhibition assay

The fungal metabolites were evaluated for their Candida Sap inhibition activity by following the method of Sundararaman et al. (2013)^74^. Fresh active cultures of *C. albicans* were inoculated (100 µL) in Sap induction medium (20 mL) (2.0 g yeast extract, 23.4 g yeast carbon base, and 4.0 g BSA in 1 L distilled H_2_O, pH 5.0) and the mixture was treated with different concentrations (1/2 MIC, MIC, and 2MIC) of fungal metabolites under the incubation at 37 °C for 48 h with mild agitation. After that, the cell-free culture supernatant (0.1 mL) was added to a 0.9 mL medium, which contained citrate buffer (0.1 M, pH 3.2) and BSA (0.2% w/v). The mixture was incubated for 1 h at 37 °C for evaluation of inhibition of proteinase action. 1 mL of 5% (w/v) ice-cold TCA was added to the mixture to cease the ongoing reaction. The mixture was centrifuged for ten minutes at 1500 g, and the supernatant’s optical density was recorded at 280 nm using a visible UV spectrophotometer (Shimadzu UV-1700, USA). Sterile distilled water was considered as a control.

#### Detection of IC_50_ values of the artificial mixture of VOCs (A-VOC)

The artificial mixture of authenticated VOCs was made by mixing the standards of volatiles purchased from Sigma in a particular ratio (following the relative ratio of VOCs emitted from endophytic fungi TML9)- based on the GC–MS-SPME data. The artificial mixture of VOCs was poured into a pre-sterilised micro cup (4–6 mm) and placed in the center of a PDA Petri plate. Agar blocks (5 mm × 5 mm × 5 mm) with pathogenic fungal hyphae were placed on the Petri plate at a 2.5-cm distance from the VOCs containing a micro cup and wrapped with two layers of Parafilm. Mycelial growths were measured (in terms of radial growth/colony diameter in centimeters) after 48 h of incubation and continued up to 168 h. Control plates lack artificial mixtures. Tests on 2–50 µL of the artificial mixture per 50 mL of air space above the mycelial culture in the PDA plate were performed on three replicates, and IC_50_ (concentration of A-VOC needed for the 50% growth inhibition of the pathogens) values in the complete air space of the test Petri plate were calculated.

#### Effects of VOCs on the leakage of intracellular components from fungal hyphae

Leakage of necessary macro-molecules, i.e., the protein, was evaluated following the standard procedures (da Rocha et al., 2015) with some modifications^75^. 100 mL of sterile PDB medium were inoculated with a 5-mm-diameter mycelial plug of post-harvest pathogen *P. digitatum* in different Erlenmeyer flasks and incubated at 27 ± 2 °C with 160 rpm for 48 h in a BOD shaker incubator. Mycelial mass was filtered with the Whatman filter paper and washed with sterile water. 1 g of the washed mycelia was resuspended in 100-mL Erlenmeyer flasks containing 30 mL of 0.85% saline. Then, different concentrations of the Artificial-VOC (A-VOC) mixture were added to pathogen-inoculated flasks. The inoculated flasks were re-incubated, and protein leakage was detected at various intervals. At each time, 2 mL of the suspension was centrifuged at 8,000 rpm for 10 min at 4 °C to obtain mycelium-free supernatant. Protein contents were measured at 595 nm after 5 min of incubation at room temperature according to the Bradford method. Besides protein, the leakage of intracellular components with absorbance at 260 nm was evaluated following the procedures of Paul et al. (2011)^76^. In brief, 1 g of filtrated mycelia of the above said two pathogens were resuspended in 50 mL of sterile water. Then, A-VOC was added at different concentrations and incubated at various intervals. Cell-free supernatants were obtained, and OD values were measured at 260 nm.

The effect of VOCs on the cell membrane of the treated pathogens was further analyzed through scanning electron microscopy following the standard procedures. The impact of VOCs on the carbohydrate metabolism of the pathogens was also studied by calculating the specific activity of the following enzymes- FBPase (fructose-1,6-bisphosphatase), PFK (Phosphofructokinase), and ICDH (isocitrate dehydrogenase) discussed in the supplementary information.

#### Optimisation of antifungal activity of TML9

The endophytic fungus was grown on basal media with carbon and nitrogen source alterations to optimize the maximum production of volatile organic compounds. At first, a 5-mm fungal block was transferred to different media compositions like different nitrogen sources (each at 4 g L^−1^)- (1) beef extract, (2) tryptone, (3) peptone, (4) yeast extract, and different carbon sources (each at 4 g L^−1^)—(1) cellulose, (2) dextrose, (3) malt extract. Agar and salt concentrations were maintained in each case according to the modified M1-D medium^77^. The emission of trans-ocimenol (the highest occurrence among other volatiles) was assessed qualitatively due to its sweet fruity odour. A rating of 0–5 has been assigned to each media composition based on olfactory observations of VOCs made by ten different individuals^78^.

With yeast extract (4 g L^−1^) and malt extract (8 g L^−1^) added as additional components, the modified PDA medium exhibits the highest olfactory score, the highest amount of biomass production (i.e., fungal hyphae), and the highest amount of antifungal activity against *P. digitatum*. In addition to these, seven other inexpensive agri-waste products—bagasse, rice hulls, oatmeal, soybean meal, wheat husk, sesame meal, and tea seed pomace—were also investigated to see how they affected the growth of biomass and the efficacy of biofumigation in controlling *P. digitatum*. The ingredients were prepared following the procedure of Yeh et al. (2020) with some modifications^53^. In brief, 5 g (for 50 mL PDA) of each ingredient was sterilised individually by autoclaving and 3.25% NaOCl treatment. They were dried, and mycelial plugs of TML9 were inoculated to each medium. their ability to inhibit the growth of *P. digitatum* was evaluated by the split plate method.

#### Bio-fumigant characters of T9-WH on control of green mold infections in oranges

From the above experiments, wheat husk was the most suitable ingredient for the maximum bio-fumigation/ antifungal activity against *P. digitatum*. Therefore, wheat husk was used to grow TML9 for in-vivo biofumigation assay. Wheat husk inoculated with TML9 (T9-WH) was produced by mixing 25 g of sterilised wheat husk with 100 mL of sterile modified PDA, and three to five of 5–8 mm mycelial discs of TML9 were inoculated. T9-WH was incubated for 12–15 days for optimum growth at 28 ± 2 °C. Then, T9-WH was air-dried and mixed with new sterile wheat husk at a 2:3 ratio (w/w). Finally, the newly inoculated T9-WH was packed in cotton bags and anti-*P. digitatum* activity was evaluated in green mold-infected oranges.

In brief, thirty organic oranges- *Citrus sinensis* (120–150 g) were obtained, and disinfected by a series of steps- first immersing at 70% ethanol for 1 min and then in—2.5% NaOCl for 3 min, again washed in 70% ethanol for 30 s, finally washed with sterilised distilled water and at last dried at laminar airflow. Five groups were made, and each group contained six oranges and was kept in sterilised transparent plastic boxes (volume 500 mL, length = 12.5 cm, h = 8.5 cm). Group-I- Only oranges without any infection or fumigation of endophyte-emitted VOCs. Group II- contains *P. digitatum*-infected oranges (as positive control), Group III-treatment group- *P. digitatum* infected and fumigated (by endophytic VOCs) oranges, Group IV- Not infected with *P. digitatum*, rather sterile saline water was inoculated onto the oranges, Group V- *P. digitatum*-infected fruits were provided with a fungicide treatment. Systemic imidazole (trade name- Syngenta Amistar Fungicide) at 2 ml L^−1^ dosage was sprayed over the infected portions. Fruits were inoculated by wounding method using a sterile needle, and wounds were inoculated with 10 µL of *P. digitatum* conidial suspension (10^6^ conidia mL^−1^). In group II (i.e., treatment group)- T9-WH (VOC emitting fungi TML9 cultured in wheat husk medium) was placed inside the square box and all the boxes of each group were sealed with Scotch® tape and incubated at 24 ± 2 °C. The development of phytopathogenic fungus was monitored daily, and after almost 6 days, the infection spread completely and drastically damaged the fruit texture. The ability of the endophytic VOCs was evaluated by comparing the disease occurrence in VOC-treated and untreated groups^79^. The experiments were repeated twice. The quality of the VOC-treated fruits was checked by measuring total sugar contents, pH, ascorbic acid, and soluble solids following the standard protocols of AOAC (2002).

### Isolation of anti-fungal metabolites and characterization of those bioactive compounds

#### Extraction of antifungal metabolites from the culture broth

TML3 was found to be the most potent antifungal producer and this was further cultured in bulk amounts to obtain the bioactive compounds for further assessments. The fungal hyphal blocks were transferred to 1.5 L of potato dextrose broth in a 2 L bioreactor (Eyela, Japan) and were fermented for 5 days under agitation at 120 rpm. The fungal culture broth was filtered using Whatman filter paper no 4. The cell-free culture extract was mixed with a double volume of ethyl acetate (EA) and stirred overnight using a magnetic stirrer. The EA part was separated using a separating funnel and was subjected to evaporation using a Rotary evaporator under low pressure and temperature. The remnants were dissolved in Dimethyl sulfoxide (DMSO) and stored for further bioactivity studies.

#### TLC and GC–MS analysis

Partial purification of the bioactive metabolites of the TML3 extract was performed using thin-layer chromatographic techniques. The dried fungal extract was re-suspended in EA, maintaining a concentration of 20 mg mL^−1^, and 10 µL of EA extract was loaded onto alumina silica TLC plates (MERCK Silica gel F254) using capillary glass tubes. Ethyl acetate and chloroform, in a ratio of 1:1, were used as running solvents, and the retention factor was calculated for all the bands under UV light. The bioactive compounds corresponding to each band were scratched, collected, and finally dissolved in 1 mL of EA, which was centrifuged (7000 rpm for 15 min) and evaporated to dryness. The dried components were then dissolved in DMSO at 100 µg mL^−1^ concentration, and antibacterial action was measured by disk diffusion technique^80^.

The active fractions obtained from TLC analysis were analysed using Gas Chromatography (TRACE 1300)-Mass (ISQ QD Single Quadrupole) Spectrometry (GC–MS) system- Thermo Scientific with ESI mode. The instrument was configured with a DB-5 Ultra Inert column (30 m length and 0.25 mm inner diameter) for a 22-min run of 1 μL sample (split-less flow) with an injector port and oven temperature of 240 °C and 50 °C respectively, having 10°Cmin^−1^ ramping time up to 260 °C with helium as the carrier gas. Tri-plus RSH-based automated injection was done. The flow velocity of the carrier gas was set at 1 mL min^−1^. The ionization source was kept at 250 °C with 70 eV of ionization energy and 0.1 kV ionization current. Mass fragmentation pattern was analysed by X-Calibur software. The identification of the various compounds was based on the SI and RSI values with the best-matched compound in the NIST library^81^. The VOCs were also detected using the GC–MS instrument following the standard methods^78^. These metabolites, both volatiles and non-volatiles, were tested for their cytotoxicity towards BJ fibroblast cells, as described in the supplementary section.

### Statistical analysis

All experiments were performed in triplicate, and the results are presented as means ± standard errors (SE). Data were analysed using Prism GraphPad software version 9.2.0 (332) (San Diego, CA, USA). Tukey’s Multiple Comparison tests at At, P < 0.05 level have been performed to detect the statistical difference.

### Ethical disclosure

Plant parts were collected, analysed, and experimental procedures on plant materials were performed following the Institutional (Vidyasagar University) Ethical Guidelines.

**Supplementary information**- The data related to the cytotoxicity analysis of the endophytic fungal metabolome, in vivo analysis of anti-Candida albicans activity, evaluation of the quality of the VOC-treated fruits, SEM images of VOC-treated hyphae, impact of VOCs on the carbohydrate metabolism are presented here.

### Supplementary Information

Supplementary Information 1.

Supplementary Information 2.

Supplementary Information 3.

Supplementary Information 4.

Supplementary Information 5.

Supplementary Information 6.

## Data Availability

The datasets generated and/or analysed during the current study are available in the NCBI-GenBank repository [Accession Number ON505944 and ON597435].

## References

[1] Ogunnigbo O, Nabiryo M, Atteh M, Muringu E, Olaitan OJ, Rutter V, Ashiru-Oredope D (2022). Exploring the antimicrobial stewardship educational needs of healthcare students and the potential of an antimicrobial prescribing app as an educational tool in selected African countries. Antibiotics.

[2] Baddley JW, Stroud TP, Salzman D, Pappas PG (2001). Invasive mold infections in allogeneic bone marrow transplant recipients. Clin. Infect. Dis..

[3] Roemer T, Krysan DJ (2014). Antifungal drug development: challenges, unmet clinical needs, and new approaches. Cold Spring Harb. Perspect. Med..

[4] Pappas PG (2003). A prospective observational study of candidemia: epidemiology, therapy, and influences on mortality in hospitalized adult and pediatric patients. Clin. Infect. Dis..

[5] Sardi JCO, Scorzoni L, Bernardi T, Fusco-Almeida AM, Mendes Giannini MJS (2013). Candida species: current epidemiology, pathogenicity, biofilm formation, natural antifungal products and new therapeutic options. J. Med. Microbiol..

[6] Seneviratne CJ, Rosa EAR (2016). Editorial: Antifungal drug discovery: New theories and new therapies. Front. Microbiol..

[7] Perfect JR (2017). The antifungal pipeline: A reality check. Nat. Rev. Drug Discov..

[8] Kachroo AH (2015). Systematic humanization of yeast genes reveals conserved functions and genetic modularity. Science.

[9] Chatterjee S, Ghosh R, Mandal NC (2020). Inhibition of biofilm- and hyphal- development, two virulent features of *Candida albicans* by secondary metabolites of an endophytic fungus *Alternaria tenuissima* having broad spectrum antifungal potential. Microbiol. Res..

[10] Fanning S, Mitchell AP (2012). Fungal biofilms. PLoS Pathog..

[11] Cragg GM, Newman DJ (2013). Natural products: A continuing source of novel drug leads. Biochim. Biophys. Acta Gen. Subj..

[12] Arora, P., Ahmad, T., Farooq, S. & Riyaz-Ul-Hassan, S. Endophytes: A hidden treasure of novel antimicrobial metabolites. In *Antibacterial Drug Discovery to Combat MDR* 165–192 (Springer Singapore, 2019). 10.1007/978-981-13-9871-1_8.

[13] Schulz B, Wanke U, Draeger S, Aust H-J (1993). Endophytes from herbaceous plants and shrubs: effectiveness of surface sterilization methods. Mycol. Res..

[14] Santra HK, Banerjee D (2022). Production, optimization, characterization and drought stress resistance by β-glucan-rich heteropolysaccharide from an endophytic fungi *Colletotrichum alatae* LCS1 isolated from clubmoss (*Lycopodium clavatum*). Front. Fungal Biol..

[15] Santra HK, Maity S, Banerjee D (2022). Production of bioactive compounds with broad spectrum bactericidal action, bio-film inhibition and antilarval potential by the secondary metabolites of the endophytic fungus *Cochliobolus* sp. APS1 isolated from the Indian medicinal herb *Andrographis paniculata*. Molecules.

[16] Rai N, Gupta P, Verma A, Tiwari RK, Madhukar P, Kamble SC, Kumar A, Kumar R, Singh SK, Gautam V (2023). Ethyl acetate extract of *Colletotrichum gloeosporioides* promotes cytotoxicity and apoptosis in human breast cancer cells. ACS Omega.

[17] Santra HK, Banerjee D (2022). Production, optimisation and evaluation of plant growth promoting abilities of heteropolysaccharides isolated from endophytic fungi
* Mucor
* sp. HELF2. Front. Microbiol..

[18] Dal’Rio I, Mateus JR, Seldin L (2022). Unraveling the *Tropaeolum majus* L. (Nasturtium) root-associated bacterial community in search of potential biofertilizers. Microorganisms.

[19] Gasparotto Junior A (2011). Antihypertensive effects of isoquercitrin and extracts from *Tropaeolum majus* L.: Evidence for the inhibition of angiotensin converting enzyme. J. Ethnopharmacol..

[20] Jakubczyk K, Janda K, Watychowicz K, Lukasiak J, Wolska J (2018). Garden nasturtium (*Tropaeolum majus* L.)-a source of mineral elements and bioactive compounds. Roczniki Państwowego Zakładu Higieny.

[21] Pena LC, Jungklaus GH, Savi DC, Ferreira-Maba L, Servienski A, Maia BHLNS, Annies V, Galli-Terasawa LV, Glienke C, Kava V (2019). *Muscodor brasiliensis* sp. Nov. produces volatile organic compounds with activity against *Penicillium digitatum*. Microbiol. Res..

[22] Baiome BA, Ye X, Yuan Z, Gaafar YZA, Melak S, Cao H (2022). Identification of volatile organic compounds produced by *Xenorhabdus indica* strain AB and investigation of their antifungal activities. Appl. Environ. Microbiol..

[23] Chatterjee S, Kuang Y, Splivallo R, Chatterjee P, Karlovsky P (2016). Interactions among filamentous fungi *Aspergillus **niger*, *Fusarium verticillioides* and *Clonostachys rosea*: fungal biomass, diversity of secreted metabolites and fumonisin production. BMC Microbiol..

[24] Li J, Fei Gu, Runian Wu, Yang JinKui, Zhang K-Q (2017). Phylogenomic evolutionary surveys of subtilase superfamily genes in fungi. Sci. Rep..

[25] Peng Y, Li SJ, Yan J, Tang Y, Cheng JP, Gao AJ, Yao X, Ruan JJ, Xu BL (2021). Research progress on phytopathogenic fungi and their role as biocontrol agents. Front. Microbiol..

[26] Sugden R, Kelly R, Davies S (2016). Combatting antimicrobial resistance globally. Nat. Microbiol..

[27] Santra HK, Banerjee D (2022). Bioactivity study and metabolic profiling of *Colletotrichum alatae* LCS1, an endophyte of club moss *Lycopodium clavatum* L. PLoS ONE.

[28] Biswas K, Bhattarcharya D, Saha M, Mukherjee J, Karmakar S (2022). Evaluation of antimicrobial activity of the extract of *Streptomyces euryhalinus* isolated from the Indian Sundarbans. Arch Microbiol.

[29] Kupferwasser LI (2003). Salicylic acid attenuates virulence in endovascular infections by targeting global regulatory pathways in *Staphylococcus aureus*. J. Clin. Investig..

[30] Schaller M (1999). Secreted aspartic proteinase (Sap) activity contributes to tissue damage in a model of human oral candidosis. Mol. Microbiol..

[31] Borg M, Rüchel R (1988). Expression of extracellular acid proteinase by proteolytic *Candida* spp. during experimental infection of oral mucosa. Infect. Immun..

[32] Chatterjee S, Ghosh S, Mandal NC (2022). Potential of an endophytic fungus *Alternaria tenuissima* PE2 isolated from *Psidium guajava* L. for the production of bioactive compounds. South Afr. J. Bot..

[33] Santra HK, Banerjee D (2022). Broad-spectrum antimicrobial action of cell-free culture extracts and volatile organic compounds produced by endophytic fungi *Curvularia eragrostidis*. Front. Microbiol..

[34] Chatterjee S, Ghosh R, Mandal NC (2019). Production of bioactive compounds with bactericidal and antioxidant potential by endophytic fungus *Alternaria alternata* AE1 isolated from *Azadirachta indica* A. Juss. PLoS ONE.

[35] Cavalheiro M, Teixeira MC (2018). Candida biofilms: Threats, challenges, and promising strategies. Front. Med..

[36] Chowdhury S, Ghosh S, Gond SK (2022). Anti-MRSA and clot lysis activities of *Pestalotiopsis microspora* isolated from *Dillenia pentagyna* Roxb. J. Basic Microbiol..

[37] Khan MS, Ahmad I, Cameotra S (2013). Phenyl aldehyde and propanoids exert multiple sites of action towards cell membrane and cell wall targeting ergosterol in *Candida albicans*. AMB Express.

[38] Ghannoum MA, Rice LB (1999). Antifungal agents: Mode of action, mechanisms of resistance, and correlation of these mechanisms with bacterial resistance. Clin. Microbiol. Rev..

[39] Zhou Y (2018). ERG3 and ERG11 genes are critical for the pathogenesis of *Candida albicans* during the oral mucosal infection. Int. J. Oral Sci..

[40] Bashir A (2022). A secondary metabolite of *Cercospora* sp., associated with *Rosa damascena* Mill., inhibits proliferation, biofilm production, ergosterol synthesis and other virulence factors in *Candida albicans*. Microb. Ecol..

[41] Holmes GJ, Clark CA (2002). First report of *Geotrichum candidum* as a pathogen of sweetpotato storage roots from flooded fields in North Carolina and Louisiana. Plant Dis..

[42] Williamson B, Tudzynski B, Tudzynski P, van Kan JAL (2007). *Botrytis cinerea*: the cause of grey mould disease. Mol. Plant Pathol..

[43] Verma R (1996). Biology and control of *Rhizoctonia solani* on rapeseed: A review. Phytoprotection.

[44] Agrios GN (2005). Plant Pathology.

[45] Michielse CB, Rep M (2009). Pathogen profile update: *Fusarium oxysporum*. Mol. Plant Pathol..

[46] Meena M, Gupta SK, Swapnil Z, A., Dubey, M. K., and Upadhyay, R. S.  (2017). Alternaria toxins: Potential virulence factors and genes related to pathogenesis. Front. Microbiol..

[47] Dagenais TRT, Keller NP (2009). Pathogenesis of *Aspergillus fumigatus* in Invasive Aspergillosis. Clin. Microbiol. Rev..

[48] Banerjee D (2010). *Muscodor albus* strain GBA, an endophytic fungus of *Ginkgo biloba* from United States of America, produces volatile antimicrobials. Mycology.

[49] Banerjee D, Pandey A, Jana M, Strobel G (2014). *Muscodor albus* MOW12 an endophyte of *Piper nigrum* L. (Piperaceae) collected from North East India produces volatile antimicrobials. Indian J. Microbiol..

[50] Singh SK (2011). An endophytic *Phomopsis* sp. possessing bioactivity and fuel potential with its volatile organic compounds. Microb. Ecol..

[51] Strobel G (2011). An endophytic/pathogenic *Phoma* sp. from creosote bush producing biologically active volatile compounds having fuel potential. FEMS Microbiol. Lett..

[52] Saxena S, Strobel GA (2021). Marvellous *Muscodor* spp.: Update on their biology and applications. Microb. Ecol..

[53] Yeh CC, Wang CJ, Chen YJ, Tsai SH, Chung WH (2021). Potential of a volatile-producing endophytic fungus *Nodulisporium* sp. PDL-005 for the control of *Penicillium digitatum*. Biol. Control.

[54] Oonmetta-Aree J, Suzuki T, Gasaluck P, Eumkeb G (2006). Antimicrobial properties and action of galangal (*Alpinia galanga* Linn.) on *Staphylococcus aureus*. LWT Food Sci. Technol..

[55] Bruce A, Stewart D, Verrall S, Wheatley RE (2003). Effect of volatiles from bacteria and yeast on the growth and pigmentation of sapstain fungi. Int. Biodeterior Biodegrad..

[56] Ye X, Chen Y, Ma S, Yuan T, Wu Y, Li Y, Zhao Y, Chen S, Zhang Y, Li L, Li Z, Huang Y, Cao H, Cui Z (2020). Biocidal effects of volatile organic compounds produced by the myxobacterium *Corrallococcus* sp. EGB against fungal phytopathogens. Food Microbiol..

[57] Bassam SE, Benhamou N, Carisse O (2002). The role of melanin in the antagonistic interaction between the apple scab pathogen *Venturia inaequalis* and *Microsphaeropsis ochracea*. Can J Microbiol.

[58] Gupta S, Kaul S, Singh B, Vishwakarma RA, Dhar MK (2016). Production of gentisyl alcohol from *Phoma herbarum* endophytic in *Curcuma longa* L. and its antagonistic activity towards leaf spot pathogen *Colletotrichum gloeosporioides*. Appl. Biochem. Biotechnol..

[59] Gupta S, Choudhary M, Singh B, Singh R, Dhar MK, Kaul S (2022). Diversity and biological activity of fungal endophytes of *Zingiber officinale* Rosc. with emphasis on *Aspergillus terreus* as a biocontrol agent of its leaf spot. Biocatal. Agric. Biotechnol..

[60] Sharma S, Gupta S, Dhar MK, Kaul S (2018). Diversity and bioactive potential of culturable fungal endophytes of medicinal shrub *Berberis aristata* DC.: A first report. Mycobiology.

[61] White TJ, Bruns T, Lee S, Taylor J, Innis MH, Gelfand DH, Sninsky JJ, White TJ (1990). Amplification and direct sequencing of fungal ribosomal RNA genes for phylogenetics. PCR Protocols: A Guide to Methods and Applications.

[62] Bauer AW, Kirby WM, Sherris JC, Turck M (1966). Antibiotic susceptibility testing by a standardized single disk method. Am. J. Clin. Pathol..

[63] Burton K (1956). A study of the conditions and mechanism of the diphenylamine reaction for the colorimetric estimation of deoxyribonucleic acid. Biochem. J..

[64] Lowry OH, Rosenbrough NJ, Farr AL, Randall RJ (1951). Protein measurement with the Folin phenol reagent. J. Biol. Chem..

[65] Mandal NC, Chakrabartty PK (1993). Succinate- mediated catabolic repression of enzymes of glucose metabolism in root- nodule bacteria. Curr. Microbiol..

[66] Ibrahim HR, Sugimoto Y, Aoki T (2000). Ovotransferrin antimicrobial peptide (OTAP-92) kills bacteria through a membrane damage mechanism. Biochim. Biophys. Acta.

[67] Carlisle PL, Kadosh D (2010). *Candida albicans* Ume6, a filament-specific transcriptional regulator, directs hyphal growth via a pathway involving Hgc1 cyclin-related protein. Eukaryot Cell.

[68] Sun L, Liao K, Wang D (2015). Effects of magnolol and honokiol on adhesion, yeast-hyphal transition, and formation of biofilm by *Candida albicans*. PLoS ONE.

[69] Lorian V (2005). Antibiotics in Laboratory Medicine.

[70] Jadhav S, Shah R, Bhave M, Palombo EA (2013). Inhibitory activity of yarrow essential oil on Listeria planktonic cells and biofilms. Food Control.

[71] Orhan G, Bayram A, Zer Y, Balci I (2005). Synergy tests by E test and checkerboard methods of antimicrobial combinations against *Brucella melitensis*. J. Clin. Microbiol..

[72] Prinsloo A, van Straten AMS, Weldhagen GF (2008). Antibiotic synergy profiles of multidrug-resistant *Pseudomonas aeruginosa* in a nosocomial environment. South. Afr. J. Epidemiol. Infect..

[73] Kaur G, Balamurugan P, Vasudevan S, Jadav S, Princy SA (2017). Antimicrobial and antibiofilm potential of acyclic amines and diamines against multi-drug resistant *Staphylococcus aureus*. Front. Microbiol..

[74] Sundararaman M, Rajesh Kumar R, Venkatesan P, Ilangovan A (2013). 1-Alkyl-(N, N-dimethylamino)pyridinium bromides: inhibitory effect on virulence factors of *Candida albicans* and on the growth of bacterial pathogens. J. Med. Microbiol..

[75] da Rocha Neto AC, Maraschin M, Di Piero RM (2015). Antifungal activity of salicylic acid against *Penicillium expansum* and its possible mechanisms of action. Int. J. Food Microbiol..

[76] Paul S, Dubey R, Maheswari D, Kang SC (2011). *Trachyspermum ammi* (L.) fruit essential oil influencing on membrane permeability and surface characteristics in inhibiting food-borne pathogens. Food Control.

[77] Pinkerton F, Strobel G (1976). Serinol as an activator of toxin production in attenuated cultures of *Helminthosporium sacchari*. Proc. Natl. Acad. Sci..

[78] Mitchell AM, Strobel GA, Moore E, Robison R, Sears J (2010). Volatile antimicrobials from *Muscodor crispans*, a novel endophytic fungus. Microbiology.

[79] Park MS, Ahn JY, Choi GJ, Choi YH, Jang KS, Kim JC (2010). Potential of the volatile-producing fungus *Nodulisporium* sp. CF016 for the control of postharvest diseases of apple. Plant Pathol. J..

[80] Wang Y, Yang M-H, Wang X-B, Li T-X, Kong L-Y (2014). Bioactive metabolites from the endophytic fungus *Alternaria alternata*. Fitoterapia.

[81] Tomsheck AR (2010). *Hypoxylon* sp., an endophyte of *Persea indica*, producing 1,8-cineole and other bioactive volatiles with fuel potential. Microb. Ecol..

[82] Jemimah Naine S, Subathra Devi C, Mohanasrinivasan V, Vaishnavi B (2015). Bioactive potential of marine derived strain *Streptomyces brasiliensis* VITJS9 isolated from South East Coast of Tamil Nadu, India. Natl. Acad. Sci. Lett..

[83] Wu Y, Li X, Dong Li, Liu T, Tang Z, Lin R, Norvienyeku J, Xing M (2023). A new insight into 6-Pentyl-2H-pyran-2-one against *Peronophythora litchii* via TOR pathway. J. Fungi.

[84] Liu M, Niu Q, Wang Z, Qi H, Liang X, Gai Y, Wang B, Yin S (2023). Comparative physiological and transcriptome analysis provide insights into the inhibitory effect of 6-pentyl-2H-pyran-2-one on *Clarireedia jacksonii*. Pestic. Biochem. Physiol..

[85] Calvo H, Mendiara I, Arias E, Gracia AP, Blanco D, Venturini ME (2020). Antifungal activity of the volatile organic compounds produced by *Bacillus velezensis* strains against postharvest fungal pathogens. Postharvest Biol. Technol..

[86] Yang X, He G, Zhao L, Yang Y, Liu Y, Xu L, Ding Z (2012). Two novel phenethylamine alkaloids from *Streptomyces* sp. YIM10049. Nat. Prod. Commun..

[87] Balogun OS, Ajayi OS, Adeleke AJ (2017). Hexahydrofarnesyl acetone-rich extractives from *Hildegardia barteri*. J. Herbs Spices Med. Plants.

[88] Kumari S, Kumari S, Attri C, Sharma R, Kulshreshtha S, Benali T, Bouyahya A, Gürer ES, Sharifi-Rad J (2022). GC-MS analysis, antioxidant and antifungal studies of different extracts of *Chaetomium globosum* isolated from *Urginea indica*. BioMed Res. Int..

[89] Das D, Arulkumar A, Paramasivam S, Lopez-Santamarina A, del CarmenMondragon A, Lopez JMM (2023). Phytochemical constituents, antimicrobial properties and bioactivity of marine red seaweed (*Kappaphycus alvarezii*) and seagrass (*Cymodocea serrulata*). Foods.

[90] Wang YY, Zheng CZ, Wang L, Xu L (2014). Synthesis, Crystal Structure and Antibacterial Activity of 4-Hydroxy-Benzaldehyde Benzoyl Hydrazone. Advanced Materials Research.

[91] Santra HK, Banerjee D (2023). Antifungal activity of volatile and non-volatile metabolites of endophytes of *Chloranthus elatior* Sw. Front. Plant Sci..

[92] Ribeiro LS, de Souza ML, Lira JMS, Schwan RF, Batista LR, Silva CF (2023). Volatile compounds for biotechnological applications produced during competitive interactions between yeasts and fungi. J. Basic Microbiol..

[93] Guluma T, Babu N, Teju E, Dekebo A (2020). Phytochemical investigation and evaluation of antimicrobial activities of Brucea antidysenterica leaves. Chem. Data Collect..

[94] Karrouchi K, Radi S, Ramli Y, Taoufik J, Mabkhot YN, Al-Aizari FA, Ansar M (2018). Synthesis and pharmacological activities of pyrazole derivatives: A review. Molecules.

[95] Tahri D, Elhouiti F, Chelghoum M, Nebeg H, Ouinten M, Yousfi M (2022). Biosynthesis and biological activities of carvone and carvotanacetone derivatives. Rev. Brasil. Farmacogn..

[96] Spencer PVD, Libardi SH, Dias FFG, Oliveira WS, Thomasini RL, Godoy HT, Cardoso DR, Bogusz Junior S (2021). Chemical composition, antioxidant and antibacterial activities of essential oil from *Cymbopogon densiflorus* (Steud.) Stapf flowers. J. Essent. Oil Bearing Plants.

[97] Polatoğlu K, Karakoç ÖC, Demirci B, Başer KHC (2018). Chemical composition and insecticidal activity of edible garland (*Chrysanthemum coronarium* L.) essential oil against the granary pest *Sitophilus granarius* L. (Coleoptera). J. Essent. Oil Res..

[98] Dalli M, Azizi S, Benouda H, Azghar A, Tahri M, Bouammali B, Maleb A, Gseyra N (2021). Molecular composition and antibacterial effect of five essential oils extracted from *Nigella sativa* L. seeds against multidrug-resistant bacteria: a comparative study. Evid. Based Complement. Altern. Med..

[99] Szkudlarek M, Heine E, Keul H, Beginn U, Möller M (2018). Synthesis, characterization, and antimicrobial properties of peptides mimicking copolymers of maleic anhydride and 4-methyl-1-pentene. Int. J. Mol. Sci..

[100] Petrović J, Kovalenko V, Svirid A, Stojković D, Ivanov M, Kostić M (2022). Individual stereoisomers of verbenol and verbenone express bioactive features. J. Mol. Struct..

[101] Lira MHPD, Andrade Júnior FPD, Moraes GFQ, Macena GDS, Pereira FDO, Lima IO (2020). Antimicrobial activity of geraniol: An integrative review. J. Essent. Oil Res..

