# Supplementary material for: Antifungal activity of bio-active cell-free culture extracts and volatile organic compounds (VOCs) synthesised by endophytic fungal isolates of Garden Nasturtium
Source: Sci Rep. 2024 May 16;14:11228. doi: 10.1038/s41598-024-60948-0 (PMC11099177; doi:10.1038/s41598-024-60948-0)
Supplement: Supplementary file 2 — Supplementary Information 2. [file 41598_2024_60948_MOESM2_ESM.doc]

**Supplementary information-**


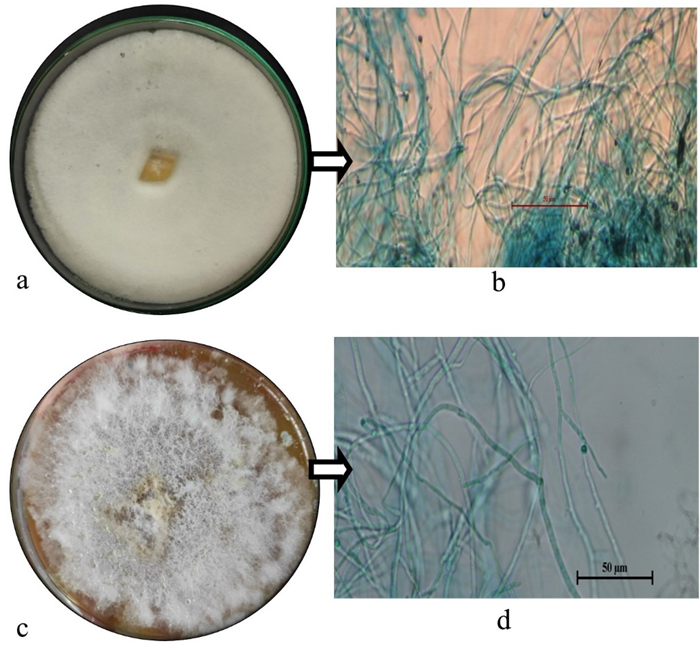


Supplementary Figure 1- Macroscopic plate morphology (Top view) of the sterile endophytic fungal isolate a- TML3 and c- TML9. Light microscopic view of the sterile hyphae of the isolates b-TML3 and d- TML9

**In vivo analysis of anti-*Candida albicans* activity of TML3 metabolites**

Methodology-

8 weeks, Male Wistar rats were injected with 100 µL of *C*. *albicans* (1x10^5^ CFU/mL) 3 h before initiating the antifungal treatment. Antifungals were given to the mice twice a day for 7 days intraperitoneally. Rats were divided into two groups, with each group having five animals. One group received 0.01, 0.1, and 10 mg/kg TML3 metabolites in 100 µL PBS, and the control group received only 100 µL PBS. The rats were weighed regularly and sacrificed after 5 days, the kidneys were taken, homogenised in PBS, and Candida load was detected by plating the liquid in SDA (Saburaud Dextrose Agar). Plates were incubated at 37°C and fungal burden was expressed as the ratio of colony forming units to the organ weight following the standard protocols of Lionakis et al. (2011).

Results-

The burden of Candida in the kidneys of TML3-treated rats was less than in the kidneys of control (non-TML3 treated) rats (Supplementary Figure 2).


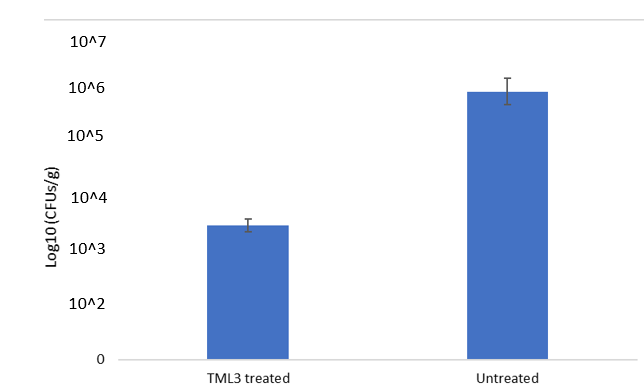


Supplementary Figure 2 Change in the load of Candida albicans in TML3-treated and untreated rats

Reference-

Lionakis MS, Lim JK, Lee CC, Murphy PM (2011) Organ-specific innate immune responses in a mouse model of invasive candidiasis. Journal of innate immunity 3: 180–199.

**In-vitro gene expression of *C*. *albicans***

Methodology-

MFC doses of TML3 metabolite were added to the *C*. *albicans* fresh culture, and another set without any TML3 metabolite served as control. Total RNA was extracted using TRIzol (Ambion, Austin, TX). DNA traces were digested by DNase I (Qiagen, Hilden, Germany) treatment. Finally, a cDNA synthesis kit (Easy script plus cDNA synthesis kit- Applied Biological Materials Inc. Richmond, BC, Canada) was used to synthesize cDNA. Next, each reaction was run with KAPA SYBR190 FAST Master Mix (KapaBiosystems, Cape Town, South Africa), and 0.2 pmol of each primer (Table 3) was added in a Stratagene Mx3000p instrument (Santa Clara, CA, USA). Amplifications were checked by the specific Tm values of the melting curves. The 2^−ΔΔCt^ method was used to estimate the relative gene expression, and the 18S rRNA gene was the endogenous control (Livak and Schmittgen, 2001).

Supplementary table 4 Oligonucleotide primers involved in the gene expression studies performed through quantitative real-time PCR.

| Gene | Sequences of the oligonucleotide primers (5'-3') | References |
| --- | --- | --- |
| *Sap1* | Forward: AACCAATAGTGATGTCAGCAGCA  Reverse: ACAAGCCCTCCCAGTTACTTTAAA | (Nailis et al., 2010) |
| *Erg3* | Forward: TGCTTCTCATGCTTTCCATC  Reverse: CCATCATGAATCATGACAGTCC | (Zhou et al., 2018) |
| *Erg11* | Forward: GAGAACGTGGTGATATTGATCC  Reverse: GAACCAAGCAGAAGTAGAAGC | (Zhou et al., 2018) |

Result-


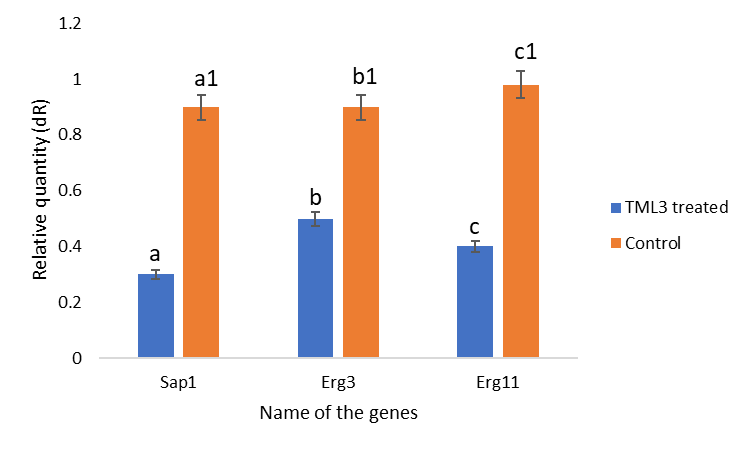
Three genes i.e., secreted aspartyl protease 1 (Sap1), sterol C5,6-desaturase (Erg3), Lanosterol 14-α-Demethylase (Erg11) involved in the degradation of immune proteins of the host (Naglik et al., 2004), and ergosterol biosynthesis (Zhou et al., 2018) of the *C*. *albicans* pathogen were (i.e., Erg3 and Erg11) down-regulated (Supplementary Figure 3) as a result of the treatment of the TML3 metabolites (MBC dose).

Supplementary Figure 3 Representation of the comparative gene expression of *C*. *albicans* treated with MBC doses of TML3 metabolites. Different letters indicate the statistical difference between the control and treated sets.

Reference-

Livak KJ, Schmittgen TD (2001) Analysis of relative gene expression data using real-time quantitative PCR and the 2− ΔΔCT method. methods 25: 402-408.484 <https://doi.org/10.1006/meth.2001>

Zhou Y, Liao M, Zhu C, Hu Y, Tong T, Peng X, Li M, Feng M, Cheng L, Ren B531 (2018) ERG3 and ERG11 genes are critical for the pathogenesis of *Candida albicans* during the oral mucosal infection. Int J Oral Sci 10: 1-8.533 <https://doi.org/10.1038/s41368-018-0013-2>

Naglik J, Albrecht A, Bader O, Hube B (2004) Candida albicans proteinases and host/pathogen interactions. Cell Microbiol 6: 915-926. <https://doi.org/10.1111/j.1462-541 5 822.2004.00439.x>

Nailis, H, Kucharíková, S, Řičicová, M, Van Dijck, P, Deforce, D, Nelis, H, & Coenye, T. (2010). Real-time PCR expression profiling of genes encoding potential virulence factors in Candida albicans biofilms: identification of model-dependent and-independent gene expression. BMC Microbiol, 10(1), 1-11. <https://doi.org/10.1186/1471-2180-10-114>

**Cytotoxicity evaluation of the endophyte synthesised metabolites**

Methodologies-

MTT-based assay-MTT (3-(4,5-dimethylthiazol-2-yl)-2,5 diphenyltetrazolium bromide) assay was adopted to evaluate the cytotoxicity of the TML’s metabolome towards rat peritoneal macrophage cells following the methodology of Mosmann (1983). In brief, different concentrations of TML3 (200-600 µg mL^-1^) and TML9 (20-70 µL 50 mL^-1^) were applied to macrophage cells, and the whole setup was cultivated overnight in RPMI-1640 supplemented with 10% FBS (Foetal Bovine Serum). The medium was changed with fresh RPMI-Roswell Park Memorial Institute (except phenol red and FBS) added with 0.5 mg/mL of MTT. A three hours incubation at a temperature of 37 ◦C was provided, and HCl- isopropanol solution was added. Next, plates were re-incubated at room temperature for 15 mins and the final absorbance (at 570 nm) of solubilised MTT formazan was measured.

SRB assay- Cytotoxicity of the TML metabolites was assessed using sulforhodamine B (SRB) assay against BJ fibroblast cell lines following the methodologies of Zhang et al. (2018). In brief, BJ cells (at a density of 8000–15000 cells/100 μL) were inoculated in 96-well plates and incubated overnight. TML metabolites were then added followed by an incubation of 48 h. Next, ice-cold trichloroacetic acid (TCA) was added as a fixative for 1 h at 4 °C. Finally, the plates were washed, and 100 µL of 0.4% SRB dye was added for 30 mins. Additional SRB dye was washed using 1% v/v acetic acid. In the next step, to completely solubilize the bound dye, 100 uL of 10 mM tris buffer (at pH 10.4) was added and shaken for 5 mins. The ultimate absorbance was measured at 540 nm on a microplate reader in triplicate. The following formula calculated the percentage cytotoxicity

Cytotoxicity= 100- [(A_1_-A_2_)/(A_3_-A_2_)] x100, here-A_1_- Absorbance value of TML metabolites, A_2_- Absorbance value of Blank, A_3_- Absorbance value of Control. Higher cytotoxicity indicates lesser cell viability and vice versa.

Results- The TML metabolites exhibited negligible cytotoxicity towards the macrophage cells and BJ fibroblast cell lines.

Supplementary Table 1-Evaluation of cytotoxicity of TML metabolites

| Concentrations (200-600 µg mL^-1^) of TML3 metabolites | Cell Viability by MTT assay | Cell Viability by SRB assay | Concentrations (µL 50 mL^-1^) of TML6 metabolites | Cell Viability by MTT assay | Cell Viability by SRB assay |
| --- | --- | --- | --- | --- | --- |
| Control (no treatment) | 100±0.17 | 100±0.15 | Control (no treatment) | 100±1.01 | 100±0.28 |
| 200 | 99.91±0.12 | 99.99±0.18 | 20 | 98.14±1.02 | 99.12±0.45 |
| 300 | 99.27±0.18 | 99.91±0.14 | 30 | 98.57±0.97 | 98.14±0.78 |
| 400 | 99.14±0.13 | 99.92±0.17 | 40 | 97.54±0.91 | 98.04±0.19 |
| 500 | 99.01±0.09 | 99.95±0.11 | 50 | 97.10±0.89 | 97.15±0.54 |
| 600 | 98.99±0.11 | 99.90±0.10 | 60 | 96.15±0.12 | 97.01±0.39 |
| 700 | 98.81±0.17 | 99.89±0.14 | 70 | 96.01±0.16 | 96.00±0.17 |

References-

Mosmann, Tim. "Rapid colorimetric assay for cellular growth and survival: application to proliferation and cytotoxicity assays." Journal of immunological methods 65, no. 1-2 (1983): 55-63.

Zhang, P.L., Wang, G., Liu, J.S., Xu, F.Q., Zhao, Z.Z., Wang, W.X., Wu, P.Y., 2018. Three new metabolites from the endophytic fungus Climacocystis montana isolated from the root bark of Paeonia ostia. Phytochem. Lett. 26, 50–54. 10.1016/j.phytol.2018.05.019


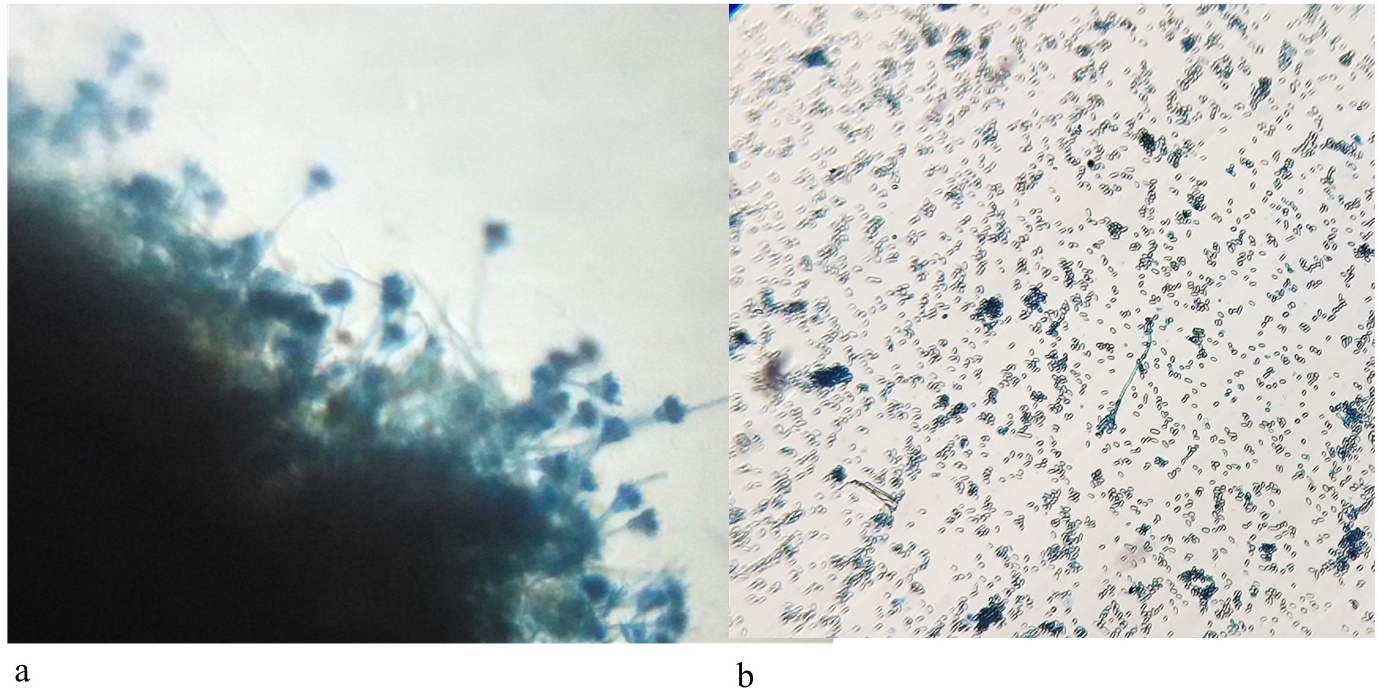


Supplementary Figure 4- a Penicillus structure of the germinated conidia in the non-VOC treated oranges, b- Less occurrence of conidial germination in TML9- VOC treated orange fruits.

Supplementary Table 2- Effect of medium compositions on VOC production by endophytic fungi TML9 with qualitative olfactory observations obtained from independent ratings of 0-5, 5 being the maximum

| **Medium composition** | **Independent olfactory score (0-5)** |
| --- | --- |
| PDA | 2 |
| CDA | 1.5 |
| Oatmeal | 1 |
| Modified PDA with instantly smashed potato + dextrose agar (M-PDA) | 2.5 |
| M-PDA + Tryptone | 3 |
| MPDA + Peptone | 3.5 |
| MPDA + Yeast extract (MPDA-YE) | 4 |
| MPDA + Beef extract | 3.5 |
| MPDA-YE + Cellulose | 4 |
| MPDA-YE + Dextrose | 4.5 |
| MPDA-YE + Malt extract | 5 |
| MPDA-YE + Sucrose | 4.5 |

**Evaluation of the impact of VOCs on the carbohydrate metabolism of the treated phytopathogens**

**Methodology-**

The three significant enzymes, FBPase (fructose-1,6-bisphosphatase), PFK (Phosphofructokinase), and ICDH (isocitrate dehydrogenase) involved in carbohydrate metabolism of the fungal cells were assayed following the methods of Mandal and Chakrabartty (1993) as described already in the manuscript.

**Results-** There was a considerable change in the enzyme profiles of the treated and untreated fungal taxa (Figure 1). The *Cercospora beticola* pathogen displays the highest degree of change in carbohydrate metabolism, followed by the other two pathogens, *Penicillium digitatum* and *Pythium ultimum*. Out of the three enzymes tested, Phosphofrutokinase was less interrupted and the phosphofructokinase was majorly inhibited.


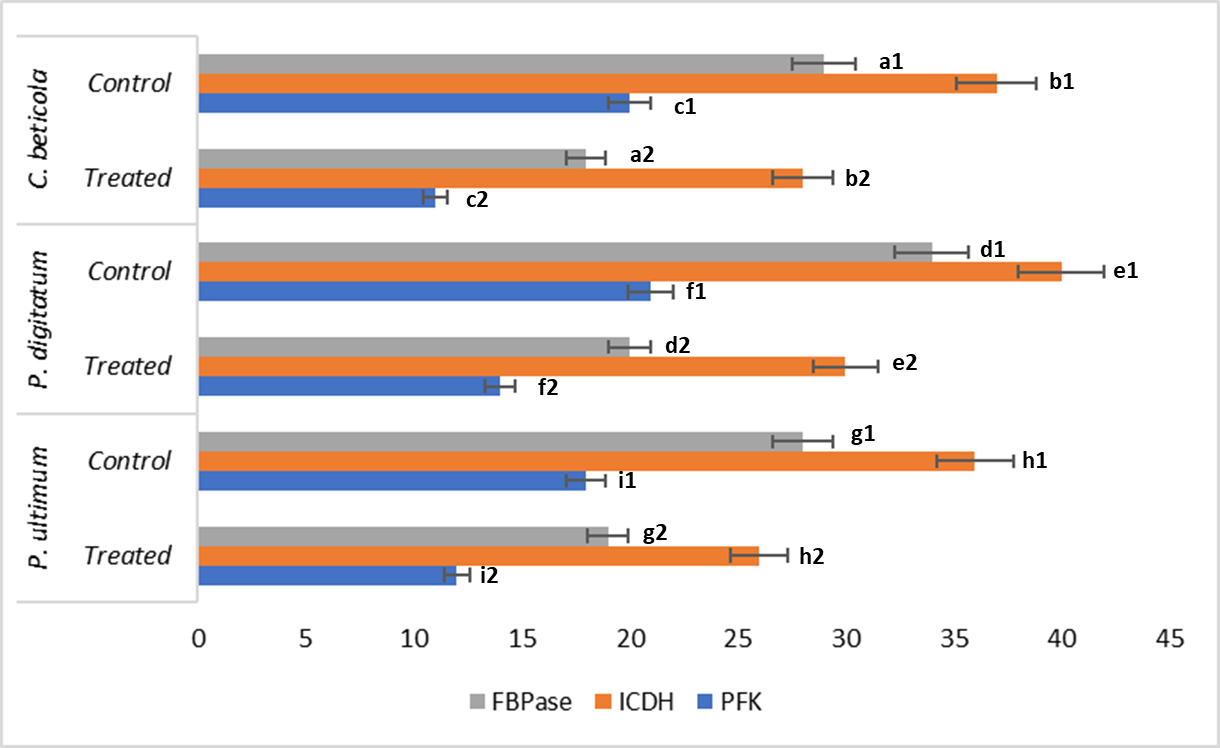


Supplementary Figure 5- Representation of the change in the profile of carbohydrate metabolizing enzymes upon treatment with TML6 metabolites. The values are presented as the mean of three individual outcomes and are expressed with standard deviation. The different letters (a_1_-a_2_, b_1_-b_2_…) on each of the control and treated sets of each enzyme for different pathogens represent valid statistical differences.

**Reference-**

Mandal NC, Chakrabartty PK. Succinate- mediated catabolic repression of enzymes of glucose metabolism in root- nodule bacteria. Curr Microbiol. 1993; 26: 247–251.

**SEM** **images of the VOC-treated hyphae of *P*. *ultimum***

Methodology-

Scanning Electron Microscopic analyses of the treated and untreated hyphae were performed following the standard protocols (Maity et al., 2020). The fungal hyphae were slowly dehydrated by dipping them in different strengths (10%-100%) of ethanol to ameliorate the chances of shrinking the mycelial structures. After that, samples were gold coated and imaged with a Zeiss EVO18 (Germany) scanning electron microscope.

Results-

The VOC-treated hyphae have lesser integrity and are of rugged appearance, whereas the non-VOC-treated ones have a normal appearance.


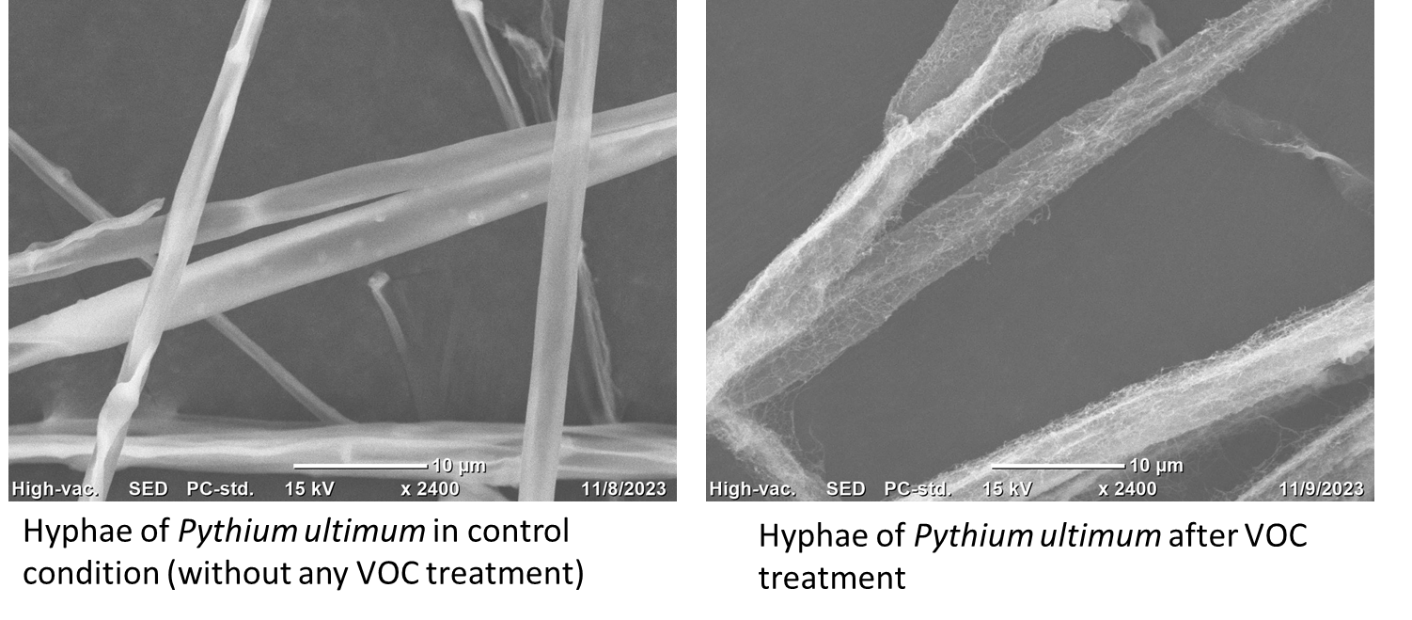


Supplementary Figure 6 SEM images of the control and VOC-treated pathogen.

Reference- Maity, S.; Mandal, S.; Santra, H.K.; Maity, S.K.; Banerjee, D. Broad-spectrum antimicrobial activity of *Streptomyces griseus* BLS4, a newly isolated endophyte of *Blumea lacera*. Res. J. Biotechnol. 2020, 15, 3


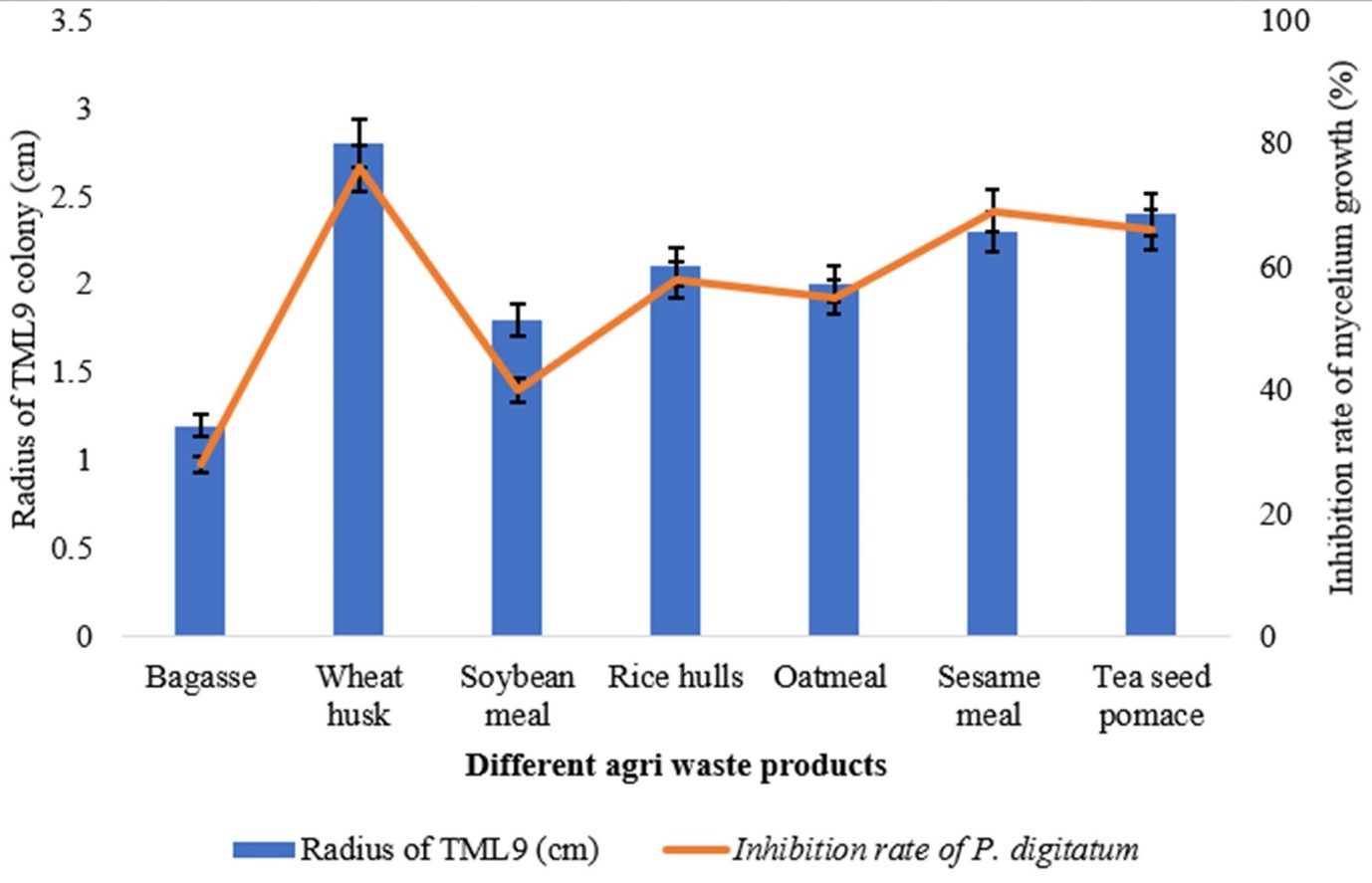


Supplementary Figure 7- Effect of different media composition on the a- growth of endophytic fungi TML9, and b- inhibition of mycelial growth of the fungal pathogen *P*. *digitatum*

**
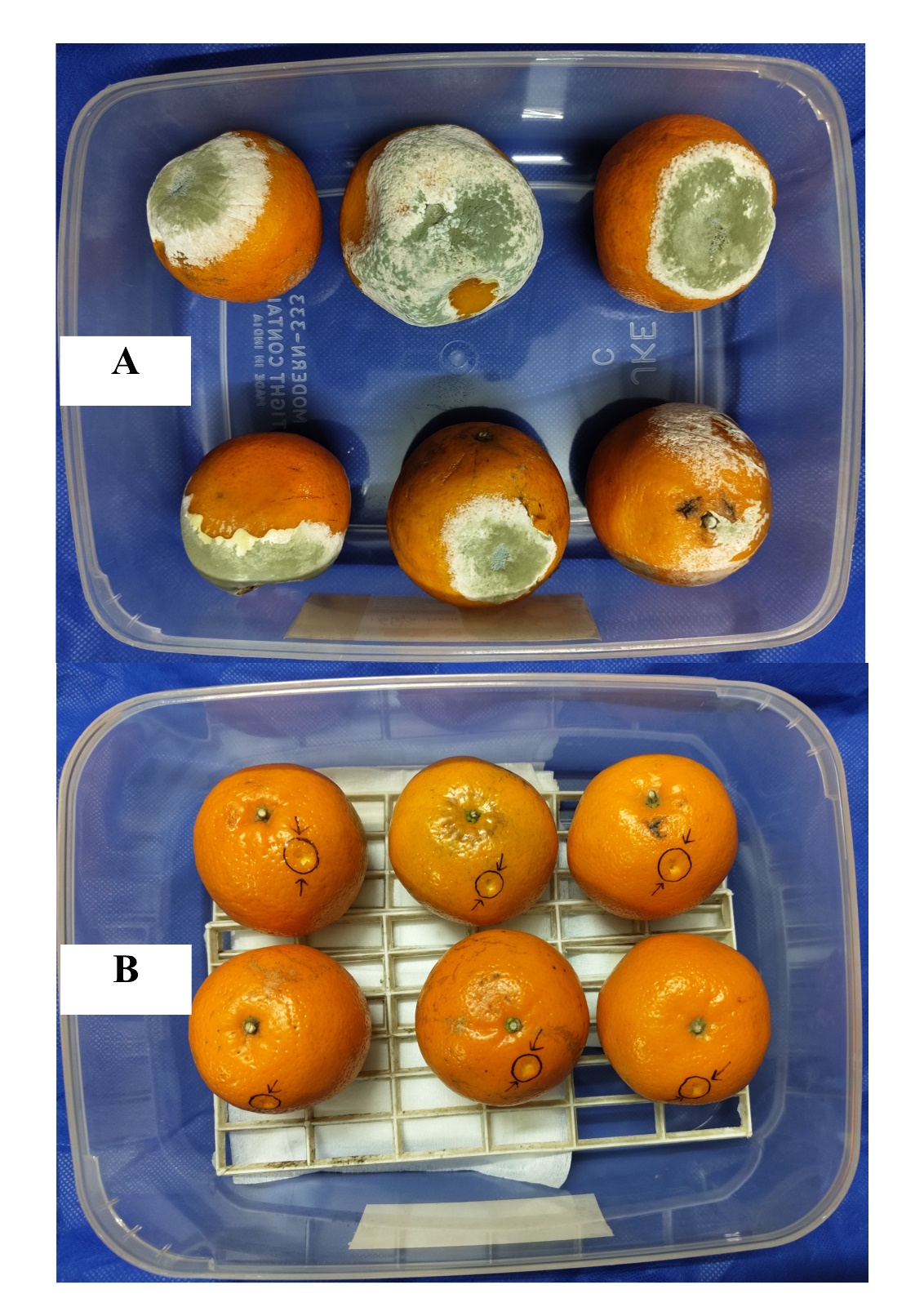
**

Supplementary Figure 8- Effect of T-WH on the control of green mold (*P*. *digitatum*) of orange. A. Inoculated by *Penicillium digitatum* without any bio-fumigation by T-WH, B. Inoculated *P*. *digitatum* and bio-fumigated by T-WH.

**Evaluation of the quality of the VOC-treated fruits**

Methodology-

Total sugar contents and soluble solids in the oranges were determined following the standard protocols of AOAC (2002). Also, ascorbic acid content was calculated following the titration method as mentioned in AOAC (2002). pH was tested after the pH meter (Sartorius PB-10) was calibrated with pH 4.0 and 7.0 phosphate buffer.

Results- The four significant parameters measured for evaluating the fruit quality are statistically similar for the VOC-treated and untreated (Supplementary Table 4). So, the VOC is non-toxic and does not hamper the standard quality of the fruit.

Supplementary Table 3 Evaluation of the fruit quality after VOC treatment

| Sl. No. | Parameters | Control | VOC treated |
| --- | --- | --- | --- |
| 1 | total sugar content (%) | 11.9±0.10^a^ | 11.7±0.14^a^ |
| 2 | pH | 4.15±0.01^b^ | 4.13±0.02^b^ |
| 3 | ascorbic acid content (mg/100g-fresh weight) | 40.17±2.43^c^ | 39.80±1.19^c^ |
| 4 | soluble solids (%) | 12.04±0.09^d^ | 11.97±0.08^d^ |

The results are the mean of three replicates of the experiments, along with the standard deviation values. The values with similar letters for each parameter in the control and VOC-treated conditions indicate statistically identical results.

Reference-

Association of Analytical Chemists, Australian Official Analytical. "Official methods of analysis of the AOAC International." (2002).
