# Supplementary figures and images for: Antifungal activity of bio-active cell-free culture extracts and volatile organic compounds (VOCs) synthesised by endophytic fungal isolates of Garden Nasturtium
Source: Sci Rep. 2024 May 16;14:11228. doi: 10.1038/s41598-024-60948-0 (PMC11099177; doi:10.1038/s41598-024-60948-0)

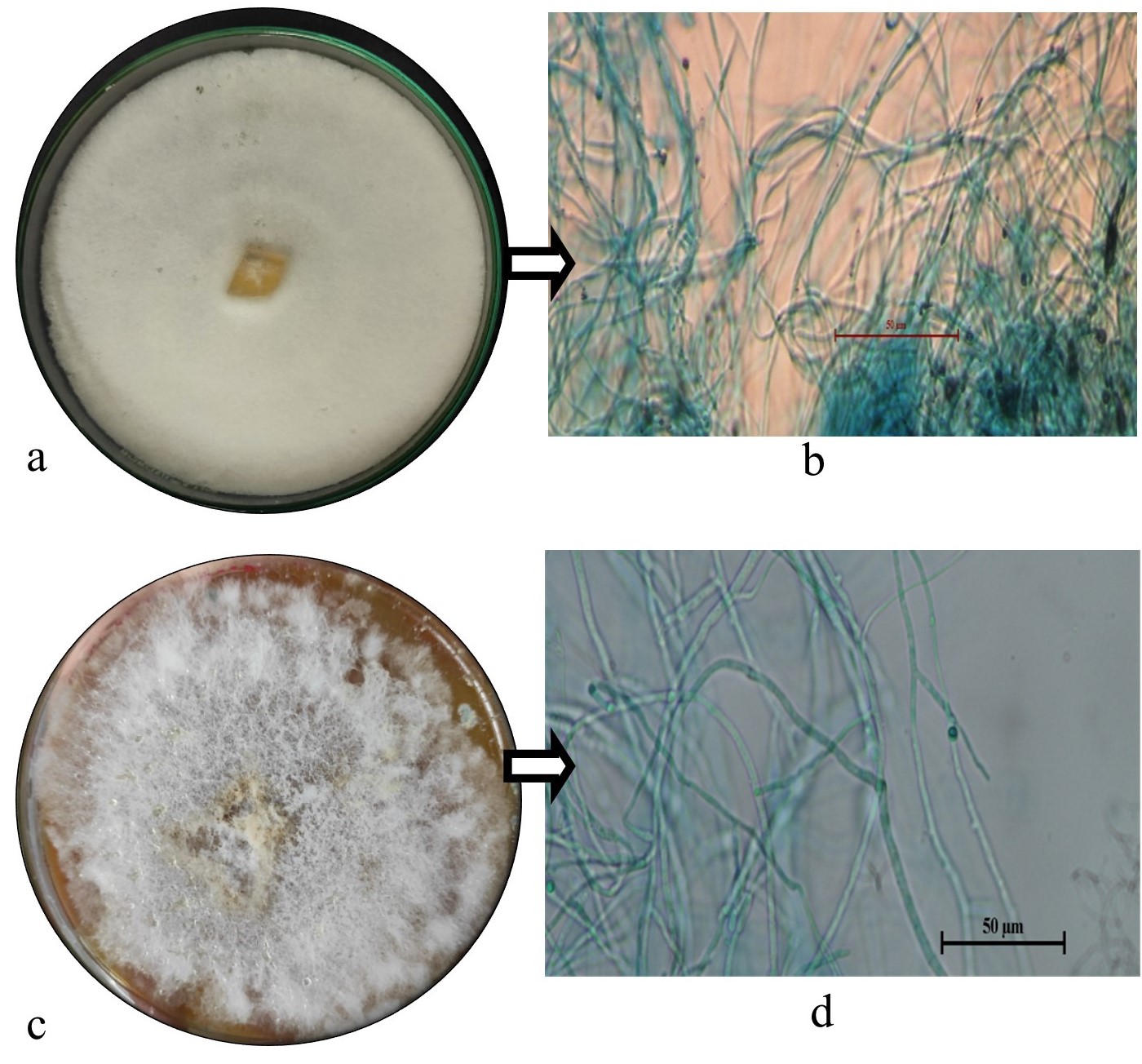

Supplement: Supplementary file 3 — Supplementary Information 3. [file 41598_2024_60948_MOESM3_ESM.jpg]

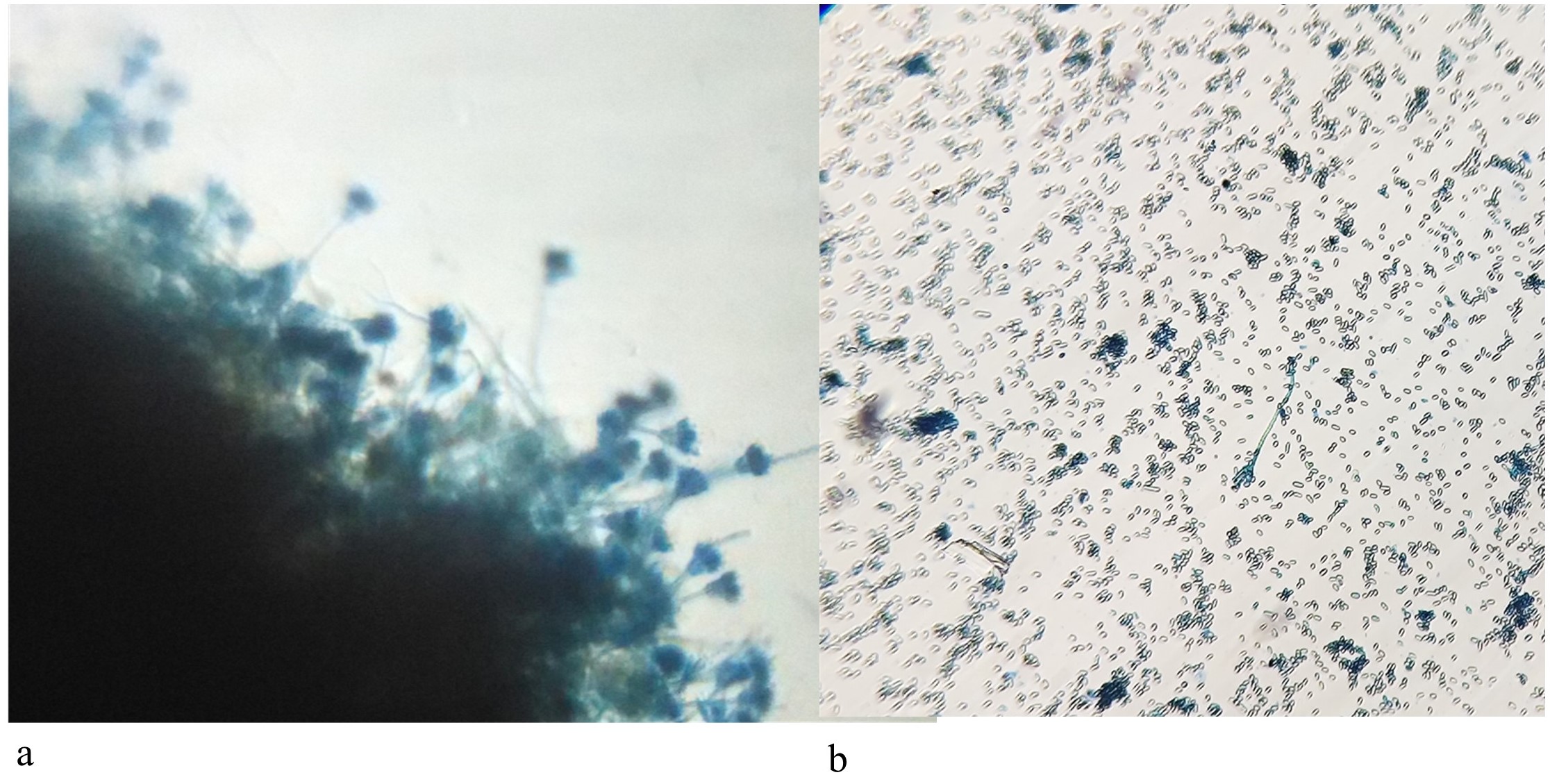

Supplement: Supplementary file 4 — Supplementary Information 4. [file 41598_2024_60948_MOESM4_ESM.jpg]

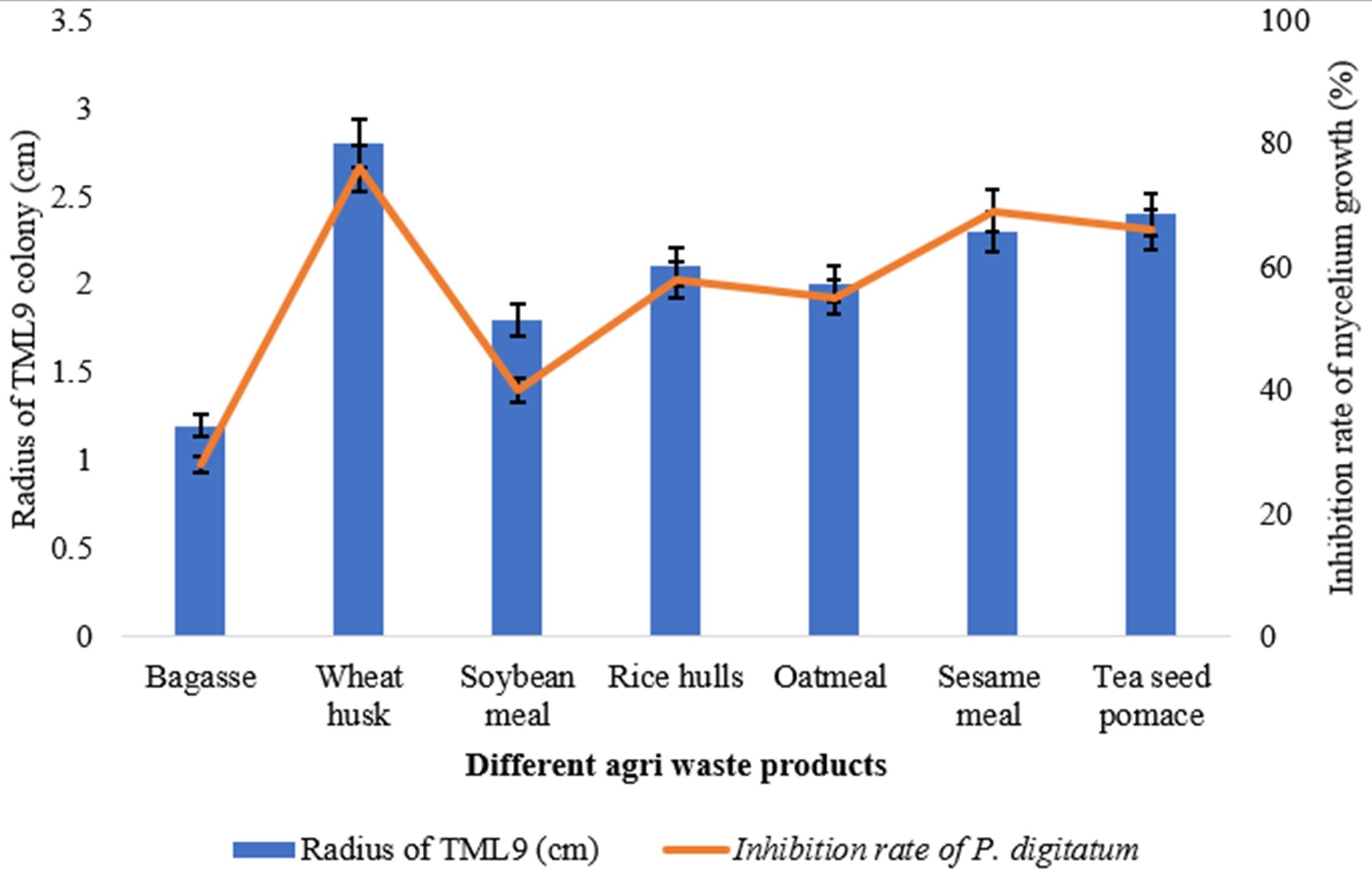

Supplement: Supplementary file 5 — Supplementary Information 5. [file 41598_2024_60948_MOESM5_ESM.jpg]

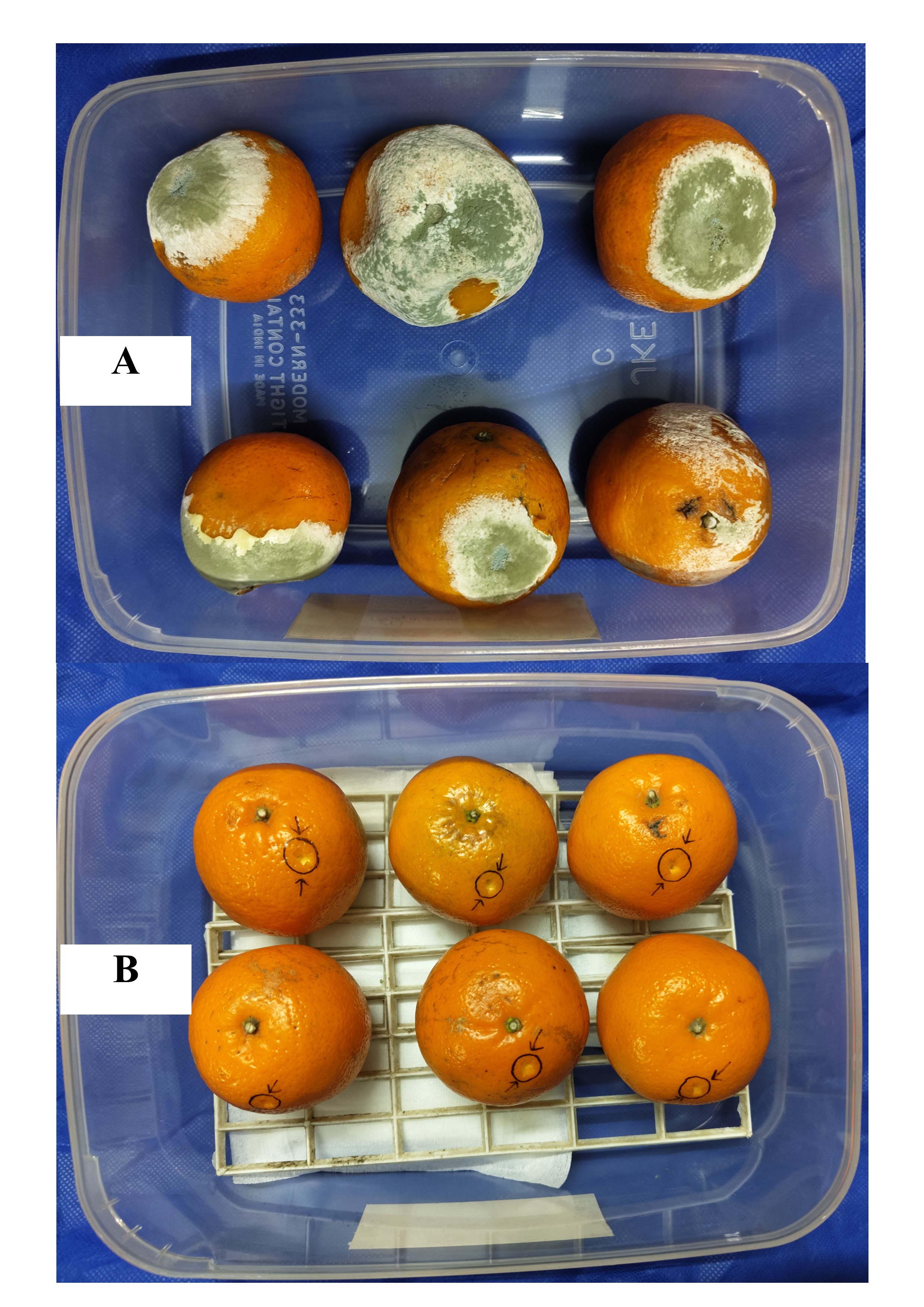

Supplement: Supplementary file 6 — Supplementary Information 6. [file 41598_2024_60948_MOESM6_ESM.jpg]
